# A systematic review of individual and community mitigation measures for prevention and control of chikungunya virus

**DOI:** 10.1371/journal.pone.0212054

**Published:** 2019-02-27

**Authors:** Catherine Hierlihy, Lisa Waddell, Ian Young, Judy Greig, Tricia Corrin, Mariola Mascarenhas

**Affiliations:** 1 Public Health Risk Sciences Division, National Microbiology Laboratory, Public Health Agency of Canada, Guelph, Ontario, Canada; 2 Department of Population Medicine, University of Guelph, Guelph, Ontario, Canada; 3 School of Occupational and Public Health, Ryerson University, Toronto, Ontario, Canada; Faculty of Science, Ain Shams University (ASU), EGYPT

## Abstract

**Background:**

Chikungunya is a mosquito-borne virus transmitted by mosquitoes from the *Aedes* genus. The virus, endemic to parts of Asia and Africa, has recently undergone an emergence in other parts of the world where it was previously not found including Indian Ocean Islands, Europe, the Western Pacific and the Americas. There is no vaccine against chikungunya virus, which means that prevention and mitigation rely on personal protective measures and community level interventions including vector control.

**Methodology/Principal findings:**

A systematic review (SR) was conducted to summarize the literature on individual and community mitigation and control measures and their effectiveness. From a scoping review of the global literature on chikungunya, there were 91 articles that investigated mitigation or control strategies identified at the individual or community level. Of these, 81 were confirmed as relevant and included in this SR. The majority of the research was published since 2010 (76.5%) and was conducted in Asia (39.5%). Cross sectional studies were the most common study design (36.6%). Mitigation measures were placed into six categories: behavioural protective measures, insecticide use, public education, control of blood and blood products, biological vector control and quarantine of infected individuals. The effectiveness of various mitigation measures was rarely evaluated and outcomes were rarely quantitative, making it difficult to summarize results across studies and between mitigation strategies. Meta-analysis of the proportion of individuals engaging in various mitigation measures indicates habitat removal is the most common measure used, which may demonstrate the effectiveness of public education campaigns aimed at reducing standing water.

**Conclusions/Significance:**

Further research with appropriate and consistent outcome measurements are required in order to determine which mitigation measures, or combination of mitigation measures, are the most effective at protecting against exposure to chikungunya virus.

## Introduction

Chikungunya virus (CHIKV) is a mosquito-borne virus that is transmitted to humans by mosquitoes from the *Aedes* genus, most commonly *Ae*. *aegypti* and *Ae*. *albopictus*. While CHIKV has been endemic in many parts of Africa and Asia for decades, it has recently re-emerged and spread to new areas including the pacific islands, South America, and the Caribbean where it was not previously recorded [1]. The large outbreak of CHIKV in the Indian Ocean Islands beginning in 2005 is believed to be due to a mutation in the East/Central/South Africa (ECSA) strain of the virus that has improved transmission by *Ae*. *albopictus* over *Ae*. *aegypti* [2]. An outbreak on Réunion Island in 2005–06 had an attack rate of 35%. Autochthonous transmission in Italy (2007), France (2010), the Caribbean islands (2013) and South America (2014) [3–6] has made CHIKV a global public health issue, as the range of affected areas continues to increase and non-endemic countries are experiencing increases in travel-related CHIKV infections [7–9]. There have also been several instances of viremic travellers importing CHIKV into regions where *Ae*. *albopictus* is present [10], which resulted in local outbreaks of CHIKV. These travel related outbreaks demonstrate that CHIKV can be imported to new areas where *Ae*. *albopictus* and *Ae*. *aegypti* are already present, which includes a high proportion of the U.S. and Europe [11, 12].

Although present in some of the same areas as dengue and malaria, CHIKV has historically received far less attention due to the self-limiting symptoms and a low risk of death. Symptoms are nonspecific and include febrile arthralgia, myalgia, headache, and rash which typically resolve within a few weeks [13], however, in a proportion of infected individuals the arthralgia is incapacitating and may lead to a chronic condition [14]. The 2005 outbreak on Réunion Island was well recorded and provides the basis for our understanding of the impact of the current CHIKV strain, including complications such as encephalitis [15], and *in utero* transmission of CHIKV [16]. The reported case fatality rate was approximately 1/1000 [17], whereas prior to the outbreak the virus was not known to cause mortality.

There is no available vaccine or antiviral treatment for chikungunya. Therefore, prevention relies primarily on individual personal protective measures and community level interventions including vector control measures. Recommendations by the Unites States Center for Disease Control and Prevention (CDC) include controlling mosquito breeding by removing stagnant water, the use of insecticides and repellents, and wearing protective clothing [18]. However, it is unclear which strategy or combination of strategies is most effective in the prevention of CHIKV.

A systematic review (SR) and meta-analysis was conducted in order to summarize which individual and community level prevention and control strategies have been investigated and which are most effective to prevent or reduce transmission of CHIKV. The results of this systematic review will highlight the consistencies and generalizability of the findings and will be useful for guiding future research and further development of educational and vector control measures to assist in reducing the risk of local transmission of CHIKV.

## Methods

### Research question, team and protocol

This SR was conducted following standard SR methodology endorsed by the Cochrane collaboration and is reported in accordance with the PRISMA guidelines [19].

The research question for this review is “what individual and community level prevention and control strategies have been investigated and which have been the most effective at reducing or preventing transmission of CHIKV”. The PICO components of this question included studies conducted on CHIKV affected human populations, examining interventions to prevent exposure to CHIKV (or exposure to mosquitoes in a CHIKV affected area) in humans, with outcomes related to the frequency of use and magnitude of effect of the mitigation strategy being examined. All controls were considered for inclusion. The review team expertise included epidemiology, public health, microbiology, vector-borne diseases, zoonotic diseases, knowledge synthesis and meta-analysis.

This SR was prioritized from a scoping review that characterized the global knowledge on CHIKV conducted by the Public Health Agency of Canada [20]. The protocol was developed *a priori* to ensure that this SR was objective, reproducible and updateable. The protocol includes the research question, definitions, inclusion criteria, and pretested tools (screening form, risk of bias tool, and the data extraction form). The protocol is available in the supplementary material (S1 Appendix).

### Scoping review search strategy, eligibility criteria and study characterisation

The scoping review search was conducted to identify all primary research related to chikungunya in English, French, Spanish, or Portuguese in seven databases: Scopus, PubMed, CINAHL, CAB, LILACS, Agricola and Cochrane. The search was conducted on May 27, 2015 and updated January 6, 2017 using the search algorithm: (Chikungunya OR CHIK OR CHIKV) OR (alphavirus AND mosquito* AND control). No date limits were applied. The scoping review included a grey literature search and an extensive search verification procedure to evaluate that relevant studies had been captured [20]. All studies on any aspect of chikungunya and its vectors were included and characterized by topic. The scoping review characterized 91 studies as examining mitigation measures at the individual or community level to prevent/control CHIKV. These 91 studies were considered for inclusion in the SR. Details of the scoping review search strategy and flow of articles are available in the supplementary material (S2 and S3 Appendices).

### Relevance screening

Primary research on the review topic in English, French, Spanish or Portuguese was eligible for inclusion. Articles were excluded if they did not contain pertinent information on mitigation or control measures. Although there are studies on the development of a CHIKV vaccine, there is currently no vaccine commercially available, and therefore studies could not be conducted on the application of a CHIKV vaccine in a community and its impact on decreasing the burden of CHIKV. For this reason articles pertaining to vaccine development were excluded. Only studies that included humans as the host species were included in this SR. Each potentially relevant study identified in the scoping review was confirmed for relevance against these eligibility criteria prior to proceeding with risk of bias assessment and data extraction. The studies included in this SR are available in the supplementary material (S4 Appendix).

### Risk of bias assessment and data extraction

The risk of bias assessment form was adapted from the tools endorsed by the Cochrane Collaboration and aimed to determine the internal validity of each study [21]. Each study was rated as having a low, high, or unclear risk of bias based on 10 criteria that appraised the study design, reporting of methodology and data exclusions. The data extraction form captured characteristics such as type and details of the intervention and data on all relevant outcomes. A low risk of bias indicates the study was done well with no concerns for biased results based on the reporting of the study, a study considered to have an unclear risk of bias indicates that one or more criteria could not be assessed due to a lack of reporting. Studies with a high risk of bias are considered to have important methodological flaws such as incomplete reporting, missing or excluded observations, inappropriate outcomes, or a lack of investigation into possible confounding variables that are likely to bias the results. All stages of the SR, relevance screening, risk of bias assessment and data extraction were completed by two independent reviewers for each study and conflicts between reviewers were resolved by consensus.

### Study management and data analysis

All stages of the scoping review and SR were conducted using the web-based management software DistillerSR (DistillerSR, Evidence Partners, Ottawa, Canada) to facilitate reviewing. Extracted data were exported to MS excel (Microsoft Corporation, Redmond, WA, USA) for data cleaning and descriptive summary. The extracted data is available in the supplementary material (S5 Appendix).

Meta-analysis was conducted using the metaprop command in STATA version 13 (StataCorp, College Station, Texas, USA). Random effects meta-analysis using the DerSimonian and Laird weighting method [22] was conducted for those mitigation measures where the proportion of the population using an intervention (prevalence) was reported. To stabilize the variances a Freeman-Tukey Double Arcsine Transformation was used when conducting the meta-analysis [23]. If the prevalence of a particular mitigation measure was reported more than once in the same study, such as during different years, they were treated as separate lines of data in the meta-analysis. The assumption of independence was not considered to be violated among these studies given these observational data represent a new sampling frame at each time period [24].

Heterogeneity was measured using *I*^2^, which describes the proportion of total variation in study estimates that is due to heterogeneity, and it was considered high if *I*^2^>50% [25]. Sub-group analysis was conducted to determine whether any of the heterogeneity between studies could be explained. The proportion of the population employing various control measures were grouped by the control method used. These groups included the following personal protective measures: use of personal repellent, room repellents, unspecified repellent use, physical barriers, habitat removal, insecticide use in or around the home, and mosquito avoidance, as well as insecticide use as a community level intervention. Within each of these subgroups we also examined whether outbreak status, whether or not an outbreak was occurring at the time of study, could be used to explain any variation in the proportion of the population using various mitigation methods.

## Results

### Descriptive statistics

**Fig 1:**
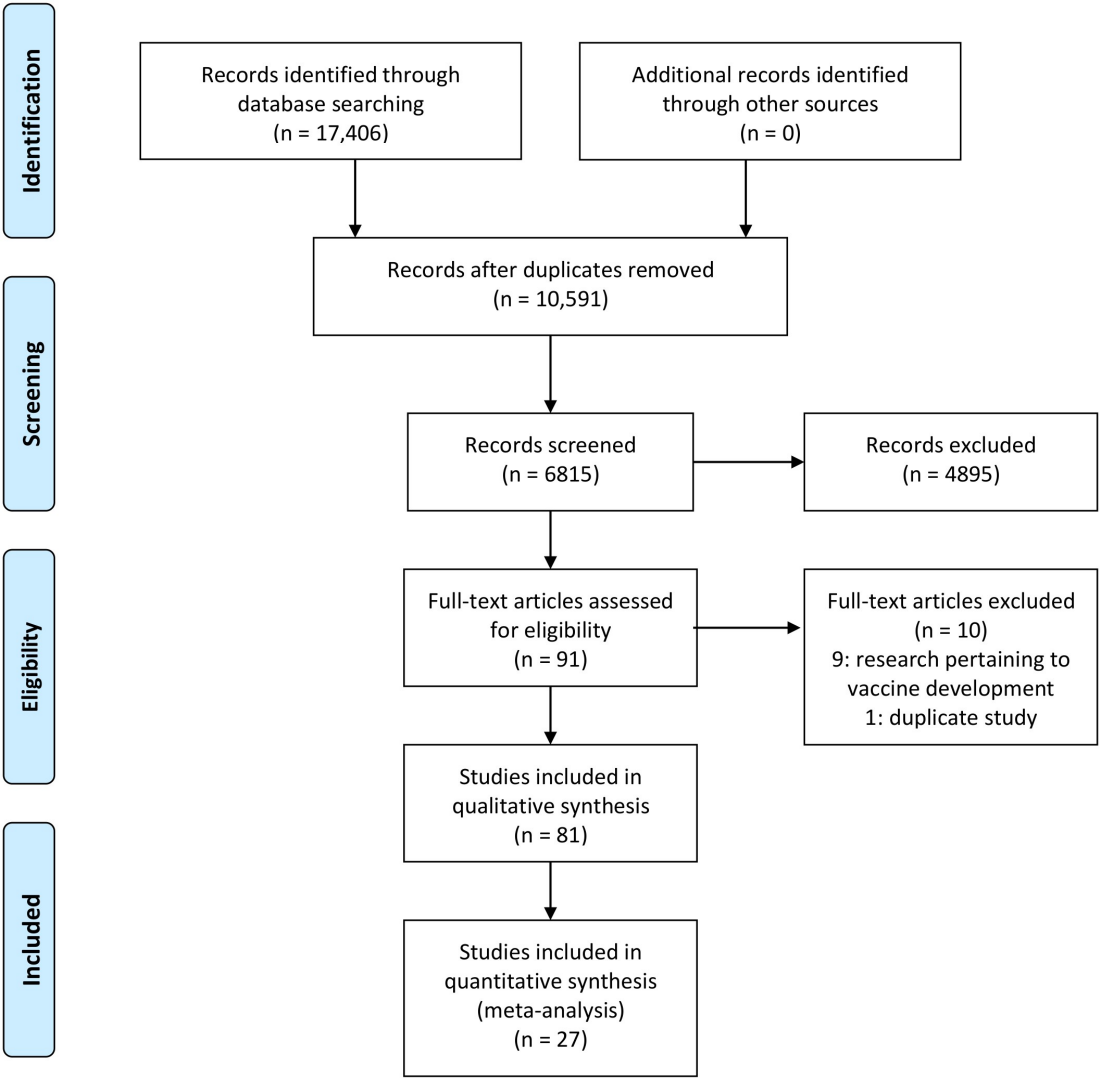
PRISMA flow diagram of articles through the scoping and systematic review processes.

There were 1920 articles characterized in the scoping review, 91 of which were identified as evaluating individual or community mitigation measures against CHIKV. Eighty-one of these studies were relevant to this SR review question, Fig 1. Of the ten excluded articles, one was excluded as a duplicate study and nine were on vaccine development for humans. The effectiveness or prevalence of the use of a vaccine in the prevention of CHIKV in a community setting was not evaluated in any study. Of the 91 included studies, 8 were disease transmission models. These studies are included in the summaries for reference as they simulate effectiveness of individual and community level control methods. However, they were not included in any further analysis as they are not considered primary research.

The 81 included studies were mainly published since 2010 (76.5%) and the largest proportion of the research originated in Asia (39.5%), while the least amount originated in Australasia (3.7%). The studies were divided into six major categories of mitigation and control measures, Table 1. The most common mitigation measures were behavioural protective measures, which were described in 67.9% of the studies. Quarantine of infected individuals was the least common measure, described in 6.2% of the studies, Table 1. Twenty-nine studies (35.8%) in this review reported on the result of mitigation measures, the majority of which (17.3%) reported on the number of chikungunya cases. Based on the risk of bias evaluation, the majority of studies scored low (51.9%) or unclear risk of bias (40.7%), whereas 8.6% were considered to have a high risk of bias, Table 1. Studies with a high risk of bias are considered to have one or more serious flaws in their conduct or reporting that may bias the reported study outcomes in an unknown direction and magnitude.

**Table 1:** General characteristics of 81 primary research publications outlining community and individual level mitigation factors for chikungunya virus.

Category		Count (percentage)
**Continent of Study** ^1^^**,**^^2^		
	Asia	32 (39.5%)
	Europe	13 (16.0%)
	Indian Ocean Islands	13 (16.0%)
	Central/ South America/Caribbean	13 (16.0%)
	North America	6 (7.4%)
	Africa	4 (4.9%)
	Australasia	3 (3.7%)
**Document Language**		
	English	77 (95.1%)
	French	2 (2.5%)
	Spanish	2 (2.5%)
**Date of Publication**		
	1971–1980	1 (1.2%)
	2001–2010	19 (23.5%)
	2011–2016	62 (76.5%)
**Risk of Bias**		
	Low risk of bias	42 (51.9%)
	Unclear risk of bias	33 (40.7%)
	High risk of bias	7 (8.6%
**Study Design**^1^		
	Observational	
	Cross sectional	30 (36.6%)
	Outbreak investigation	14 (17.1%)
	Case study/series	6 (7.3%)
	Prevalence survey	5 (6.1%)
	Cohort	4 (4.9%)
	Surveillance/monitoring program	4 (4.9%)
	Case control	1 (1.2%)
	Experimental	
	Quasi-experiment	8 (9.8%)
	Controlled trial	1 (1.2%)
	Disease transmission model	8 (9.6%)
	Descriptive	7 (8.5%)
	Risk assessment	2 (2.5%)
	Mixed methods	1 (1.2)
**Mitigation measure(s) described**^1^		
	Behavioural protective measures	55 (67.9%)
	Use of insecticides	48 (59.3%)
	Public education	31 (38.3%)
	Control/treatment of blood products	9 (11.1%)
	Biologic mosquito control	6 (7.4%)
	Quarantine of infected individual	5 (6.2%)
**Outcome measurement**^3^		
	Number of CHIKV cases	14 (17.3%)
	Density of vector population	11 (13.6%)
	Presence of breeding habitat	4 (4.9%)
	Level of knowledge	4 (4.9%)
	Presence of antibody (Ab) response to *Ae*. *albopictus* salivary proteins	2 (2.5%)

^1^ Total number sums to >81 as studies can fall into more than one category.

^2^ Total percentages do not equal 100 due to rounding.

^3^ Total number sums to <81 as not all studies reported outcomes.

### Behavioural protective measures

Behavioural protective measures were investigated in 55 studies. These include personal protective measures that individuals can apply to themselves or a living space to prevent mosquito bites such as the use of repellents (applied to the skin and in the form of diffusers), physical barriers such as long clothing, bed nets and screens, removal of vector breeding habitat, and mosquito avoidance, Table 2. Of the 55 studies, four used behavioural protective measures in disease transmission models to predict the effects of different mitigation measures [26–29].

**Table 2:** Frequency of use and effectiveness of behavioural protective measures for the prevention of chikungunya virus infection in humans.

Ref	Publication year/location	Proportion and description of protective measure	Sample size (cases/controls)^1^	Outcome
**Removal of breeding habitat**				
South/Central America				
[39]	2014/Colombia	81% removal of standing water	171	n/r^2^
[56]	2016/French Guiana	32.3% remove stagnant water	1462	n/r
[58]	2016/Nicaragua	73.1% eliminate breeding sites	848	n/r
		56.8% cover water containers	848	n/r
		38.3% clean water containers	848	n/r
Europe				
[33]	2012/France	17.7% eliminate standing water	1506	n/r
[42]	2013/Spain	Avoid stagnant water on property 58.4% (2008), 57.6% (2009), 68.7% (2010)	428 (2008), 245 (2009), 147 (2010)	n/r
Asia				
[51]	2007/India	n/r	n/r	Reduced incidence of cases and larval densities^3^
[61]	2011/India	78% cover water storage containers	50	n/r
[46]	2011/India	Changing stored water frequently: cases 44%, controls 87%	600 (150/450)	A greater proportion of CHIKV negative controls change standing water and turn over empty containers than CHIKV cases (p<0.001)
[43]	2012/China	n/r	n/r	Decreased Breteau and mosq-ovitrap indices 2 weeks following application^3^
Indian Ocean Islands				
[38]	2008/Reunion Island	57% often destroy habitat, 27% never destroy habitat	1035	Increased odds of CHIKV with habitat destruction (OR 1.12, p<0.05)
[49]	2009/Maldives	n/r	n/r	Decreased Breteau Index^3^
[35]	2009/Mayotte Island	82.7% eliminate artificial breeding sites	888	The prevalence of CHIKV was higher among individuals that did not eliminate artificial breeding sites from their property (p<0.05)
		78.3% empty water from receptacles	888	n/r
		80.4% cover or turn over storage containers	888	n/r
[62]	2010/Reunion Island	78% implemented individual protections against mosquito bites or preventive measures against breeding places	74	n/r
[37]	2014/Reunion Island	97% eliminate standing water	1029	n/r
[53]	2014/Reunion Island	49.5% prevent breeding sites on their property	850,804	n/r
**Repellents Applied to a Person**				
North America				
[66]	2016/US Virgin Islands	87% do not wear repellent treated clothing	433	n/r
		56% use skin repellents	440	n/r
[67]	2016/USA	16% use permethrin on clothing	149	n/r
Europe				
[33]	2012/France	17.4% applied repellents to skin	1506	n/r
Asia				
[34]	2010/India	17.7% use repellent applied to the skin	857	n/r
[63]	2014/India	10% use repellent creams	81	n/r
Indian Ocean Islands				
[38]	2008/Reunion Island	35.8% use repellent creams and sprays	1035	Lack of repellent use is associated with contraction of CHIKV (OR 1.4, p<0.001)
[65]	2008/Reunion Island	69.7% use skin repellent	221 cases	No significant difference in repellent use among CHIKV cases (p = 0.08)
[64]	2009/Reunion Island	79% of parents applied a repellent product more than once per day on the skin of their child	277	n/r
[37]	2014/Reunion Island	2.25% use repellent bracelets	1024	n/r
		0.10% use anti-mosquito patch	1024	n/r
		22.85% use essential oils	1024	n/r
		36.82% use anti-mosquito body sprays/creams	1024	n/r
Australasia				
[68]	2016/Australia	Use of insecticide treated clothing: cases 37.2%, controls 37.3%	102 (43/59)^4^	No difference in CHIKV infection for use of treated clothing (p = 0.99)
**Space Repellents**				
North America				
[72]	2016/Mexico	Use of citronella candles: cases 9.5%, controls 19.9%	250 (74/176)	Fewer CHIKV cases when Citronella is used (OR 0.37, 95%CI 0.12–0.99).
		Use of mosquito coils: cases 24.3%, controls 28.4%	250 (74/176)	No effect of mosquito coil use (OR 0.84, 95% CI 0.4–1.75)
Asia				
[69]	2008/India	55.1% use mosquito coils	300	n/r
		45.5% fumigated with plant based materials	300	n/r
[34]	2010/India	46.6% use fumes as a repellent	857	n/r
		15.1% use mosquito coils	857	n/r
		4% use a mosquito mat or liquidator	857	n/r
[70]	2011/India	5.7% use liquidator or mat	528	n/r
[63]	2014/India	60% use liquid vaporizers	81	n/r
		24.4% use coils	81	n/r
		17.8% use repellent mats	81	n/r
[71]	2016/Bangladesh	64% use mosquito coils	1933	Mosquito coils had no impact on transmission risk (OR 1.0, 95% CI 0.8–1.2)
Indian Ocean Islands				
[38]	2008/Reunion Island	45.5% use diffusers	1035	Lack of repellent use is associated with contraction of CHIKV (OR 1.4, p<0.001)
[37]	2014/Reunion Island	69.04% use mosquito coils	1024	n/r
		52.73% space sprays	1024	n/r
		38.09% use non-electric diffusers	1024	n/r
		19.43% use rechargeable electric vaporizers/diffusers	1024	n/r
		0.29% use ultra-sound devices	1024	n/r
		18.55% use plants (citronella, geranium)	1024	n/r
**Unspecified Repellent Use**				
North America				
[67]	2016/USA	59% use repellents in travel to CHIKV risk areas	149	n/r
[72]	2016/Mexico	Use of repellent in the last month: cases 31.1%, controls 34.1%	250 (74/176)	No effect of repellent use in the last month (OR 1.06, 95% CI 0.58–1.94)
South/Central America				
[58]	2016/Nicaragua	44.1% use repellents	848	n/r
Europe				
[48]	2010/Italy	n/r	325	Fewer cases of CHIKV among those using repellents (OR 0.35, 95% CI 0.15–0.77)
[42]	2013/Spain	Use repellents 45.8% (2008), 46.9% (2009), 15.0% (2010)	428 (2008), 245 (2009), 147 (2010)	n/r
Asia				
[73]	2010/India	58% of houses used mosquito repellent in the last month	1301	n/r
[46]	2011/India	Mosquito repellent use: cases 47%, controls 71%	600 (150/450)	Greater proportion of those negative for CHIKV used repellents than CHIKV positive cases (p<0.001)
[45]	2011/India	88.4% use repellents	354	n/r
[75]	2015/India	52.5% use repellents	135	n/r
[74]	2016/India	57.5% use repellents	247	n/r
[59]	2016/Suriname	Mean repellent use over 3 surveys 47.4%	1637, 1583, 1622	For 2 of 3 surveys a greater proportion of self-reported CHIKV positive cases used repellent than did CHIKV negative cases (p = 0.026, p = 0.01), no difference in third survey (p = 0.45)
Australasia				
[68]	2016/Australia	Use of repellents: cases 93%, controls 94.9%	102 (43/59)^4^	No difference in CHIKV infection for use of repellent (p = 0.69)
**Physical Barrier—Space**				
North America				
[66]	2016/Us Virgin Islands	92% do not use a mosquito net	433	n/r
		75% stayed in screened or air conditioned rooms	433	n/r
[67]	2016/USA	5% use mosquito nets in CHIKV risk areas	149	n/r
[72]	2016/Mexico	Use of screens: cases 62.2%, controls 55.7%	250 (74/176)	No impact on CHIKV transmission (OR 1.07, 95% CI 0.57–2.01)
		Open windows: cases 90.5%, controls 78.4%	250 (74/176)	No impact on CHIKV transmission (OR 1.49, 95% CI 0.63–3.53)
South/Central America				
[56]	2016/French Guiana	32.9% close windows	1462	n/r
[58]	2016/Nicaragua	48.5% use mosquito nets	848	n/r
		18.8% use window screens	848	n/r
Europe				
[48]	2010/Italy	n/r	325	Lower prevalence of CHIKV among those who use window screens (OR 0.43, 95% CI 0.21–0.89)
Asia				
[69]	2008/India	30% use mosquito nets	300	n/r
[34]	2010/India	2.6% use mosquito nets	857	n/r
[46]	2011/India	Use of mosquito nets: cases 33%, controls 56%	600 (150/450)	Individuals with CHIKV were less likely to use mosquito nets (p<0.001)
		Use of screens: cases 13%, controls 43%	600 (150/450)	Individuals with CHIKV were less likely to use screens (p<0.01)
[45]	2011/India	8.2% use mosquito nets	354	n/r
		8.2% use window screens	354	n/r
[63]	2014/India	2.2% use mosquito nets	81	n/r
[75]	2015/India	28.9% use mosquito nets	135	n/r
[74]	2016/India	14.2% use screens	247	n/r
		30.8% use mosquito nets	247	n/r
Indian Ocean Islands				
[38]	2008/Reunion Island	43.2% use mosquito nets	1035	n/r
[64]	2009/Reunion Island	Use of a mosquito net to protect infants under 30 months of age: overall 70%, by age <6 months 78%, 6–12 months 70%, 12–24 months 62% and >2 years 57%	277	n/r
[37]	2014/Reunion Island	14.26% use mosquito nets	1024	n/r
Australasia				
[68]	2016/Australia	Use of door screens: cases 0%, controls 0.03%	102 (43/59)^4^	No difference in CHIKV infection for use of door screens (p = 0.51)
		Use of windows screens: cases 0%, controls 0.08%	102 (43/59)^4^	No difference in CHIKV infection for use of window screens (p = 0.07)
		Use of mosquito nets: cases 95.3%, controls 96.6%	102 (43/59)^4^	No difference in CHIKV infection for use of mosquito nets (p = 0.57)
Africa				
[77]	2016/Gabon	Mosquito net use: 79–96%	162	No correlation between bed net use and CHIKV infection^3^
**Physical Barrier—Long Clothing**				
North America				
[66]	2016/US Virgin Islands	67% do not wear long clothing	432	n/r
Asia				
[46]	2011/India	Wearing long clothing: cases 0%, controls 80%	600 (150/450)	Individuals with CHIKV were less likely to wear long dresses (p<0.001)
[75]	2015/India	13.3% use protective clothing	135	n/r
Indian Ocean Islands				
[35]	2009/Mayotte Island	17.1% wear long clothing	888	n/r
Australasia				
[68]	2016/Australia	Use of protective clothing: cases 55.8%, controls 62.7%	102(43/59)	No difference in CHIKV infection for use of protective clothing (p = 0.42)
**Unspecified Barrier**				
Europe				
[42]	2013/Spain	Use of physical barriers 20.8% (2008), 15.5% (2009), 6.9% (2010)	428 (2008), 245 (2009), 147 (2010)	n/r
**Avoidance**				
[35]	2009/Mayotte Island	28.9% reduce outdoor activities	888	n/r
		50.8% avoid mosquito infested areas	888	n/r

^1^ Cases/controls = number of individual who were positive for CHIKV/ negative for CHIKV in the study.

^2^ Indicates the measure was not reported.

^3^ Extractable data was not provided in the article.

^4^ Individuals were cases if they were CHIKV or dengue positive.

Removal of vector habitat was the most commonly reported behavioural measure (33 studies) [30–62]. The proportion of the population engaging in this behaviour was reported in 12 studies [33, 35, 37–39, 42, 46, 53, 56, 58, 61, 62] and ranged from 17.7% [33] to 97% [37], Table 2. Several studies mentioned that breeding habitat removal occurred without providing any further details [30–32, 45, 48, 55, 57, 60]. Other studies mentioned removal of vector habitat such as solid waste [42], removal of bushes and grasses [52], and yard sanitation [53] or provided no details on the type of vector habitat that was removed [40, 41, 47, 50].

The effectiveness of breeding habitat removal was reported in six studies. On Mayotte Island in 2009 the prevalence of CHIKV was found to be greater in individuals who did not remove breeding habitat (chi squared p<0.05) [35], and a study in India from 2011 reported that CHIKV negative participants had an odds ratio of 6.68 (95% CI 4.16–10.74) for reporting changing stored water frequently and an odds ratio of 10.34 (95% CI 6.33–16.91) for reporting that they turn empty containers upside down [46]. Another study from India reported a decrease in the incidence of cases following removal of breeding habitat [51]. In contrast, one study reported slightly greater odds, 1.12 (p<0.05), of contracting CHIKV among individuals who reported destroying breeding sites around their home [38]. Removal of breeding habitat was also associated with a decrease in larval densities in three studies [43, 49, 51].

Mosquito repellents include synthetic and natural substances that deter mosquitoes from approaching or landing. They can be applied to an individual through the use of creams or sprays or treated clothing, or they can be used to exclude mosquitoes from a space through the use of diffusers, smoke, or ultra-sound devices. The use of mosquito repellents was reported in 30 studies. The proportion of the population using individual repellents was reported in 10 studies [33, 34, 37, 38, 63–68] and ranged from 0.1% [37] to 79% [64], Table 2. The proportion of the population using space repellents was reported in eight studies [34, 37, 38, 63, 69–72] and ranged from 0.3% using an ultra-sound device to 69% using a mosquito coil [37], Table 2. The proportion of the population using an unspecified repellent was reported in 11 studies [42, 45, 46, 58, 59, 67, 68, 72–75]. Five studies mentioned that repellents were used without providing any further details [40, 52, 56, 61, 62]. One study determined the effectiveness of various repellents by comparing the length of time they kept *Ae*. *aegypti* at bay [76].

The effectiveness of skin repellent or repellent impregnated clothing was evaluated in two studies, both of which reported no effect on the rate CHIKV infections [65, 68]. Among observational studies the lack of repellent use (unspecified type) resulted in greater odds of contracting CHIKV, OR 1.4 (p<0.001) [38], the lack of space repellent use had a greater odds of contracting CHIKV, OR 3.45 (95% CI 2.34–5.09) [46] and OR 2.85 (95% CI 0.15–0.77) [48], however two other studies found no significant association between the use of space repellents and CHIKV infection [71, 72]. Citronella use had a marginally significant protective association with CHIKV infection, OR 0.37 (95% CI 0.12–0.99) whereas there was no association between CHIKV infection and the use of mosquito coils [72].

Physical barriers were described in 26 studies and can be divided into individual barriers such as long clothing, or space barriers such as bed nets, and screens. The proportion of the population using individual physical barriers was reported in five studies [35, 46, 66, 73, 75] and ranged from 0% to 80% [46], Table 2. Six studies reported the use of mosquito nets without any further details [33, 40, 52, 54, 55, 70]. Only two studies reported outcomes on the use of individual barriers. One study found that individuals with CHIKV were less likely to wear long clothing [46], while the other found no association between CHIKV infection and wearing long clothing [68]. The proportion of the population using space barriers was reported in 17 studies [34, 37, 38, 45, 46, 56, 58, 63, 64, 66–69, 72, 74, 75, 77] and ranged from 2.2% [63] to 96.6% [68]. Most of the studies that examined outcomes of space barriers found a positive effect. One study from India in 2011 reported that CHIKV negative participants had 4.51 (95% CI 2.94–6.93) greater odds of reporting mosquito net use and 7.4 (95% CI 3.18–17.22) greater odds of using window/door screens in their homes [46]. A study from Italy in 2010 found that individuals who did not use window screens had 2.32 (95% CI 1.12–4.76) greater odds of contracting CHIKV [48]. Two studies found no association between the use of a space barrier and CHIKV infection [68, 77].

Avoidance of mosquitoes was mentioned in three studies [33, 35, 56]. Only one of the studies mentioned the proportion of the population avoiding mosquitoes by reducing outdoor activities (28.9%) or avoiding mosquito infested areas (50.8%) [35], Table 2. The others provided no further information [32, 56] and none evaluated effectiveness.

### Use of insecticides

Thirty-one studies investigated the use of insecticides as a control measure. Insecticide use consisted of space spraying with adulticides in and around homes (22 studies) or the addition of larvicides to water sources (9 studies), Table 3. Of the 31 studies, five were disease transmission models that simulated the effects of insecticide use in controlling an outbreak [26, 28, 29, 78, 79]. Another 17 studies reported on the use of insecticides without providing any further details [31, 32, 34, 36, 40, 45, 47, 50, 53, 57, 60, 75, 80–84].

**Table 3:** Frequency and effectiveness of insecticide use for the prevention and control of chikungunya virus infection in humans.

Ref	Publication year/location	Insecticide used	Prevalence of use	Sample size (cases/controls)^1^	Outcome
**Adulticide**					
Europe					
[33]	2012/France	n/r^2^	20.2% of survey respondents	1506	n/r
[42]	2013/Spain	Alfacipermetrin	16.4% (2008), 17.1% (2009), 6.1% (2010)	428 (2008), 245 (2009), 147 (2010)	n/r
[30]	2015/France	Deltamethrin	n/r	n/r	Fogging reduced the mosquito population by 97% 48hrs after treatment
South America					
[56]	2016/French Guiana	n/r	34.7% use indoor insecticide sprays	1462	n/r
Asia					
[85]	1975/Burma	Pyrethrum	n/r	n/r	Decreased house index^3^
[73]	2010/India	n/r	42.4% of households	1301	n/r
[46]	2011/India	n/r	CHIKV cases 87%, controls 97%	600 (150/450)	Greater use of insecticides among CHIKV negative controls than among CHIKV cases (p<0.001)
[70]	2011/India	n/r	16.9% of homes	528	n/r
[81]	2011/Singapore	n/r	n/r	n/r	Median larval densities in clusters dropped from 380 in 2008 to 100 in 2009, (p = 0.011)
[86]	2012/India	Pyrethroid	n/r	n/r	Decreased number of suspected cases^3^
[43]	2012/China	n/r	n/r	n/r	Decrease in Breteau and Mosq-ovitrap indices 2 weeks following application
[63]	2014/India	Sprays with pyrethrum and its related compounds	32.2% of survey respondents	81	n/r
[87]	2015/India	Pyrethrum	n/r	n/r	Decline in CHIKV cases 3 weeks following application^3^
[74]	2016/India	n/r	6.07% use insecticidal sprays	247	n/r
Indian Ocean Islands					
[38]	2008/Reunion Island	n/r	Sprays: never 32.5%, sometimes 18.1%, often 35.9%	1035	Decreased odds of contracting CHIKV when using household insecticide OR 0.83 (p<0.05)
			Diffusers: never 10.5%, sometimes 18.1%, often 71.4%	1035	Decreased odds of contracting CHIKV when using household insecticide OR 0.83 (p<0.05)
[49]	2009/Maldives	n/r	n/r	n/r	Decreased Breteau index^3^
[44]	2012/Reunion Island	Naled and Pyrethrum	n/r	n/r	Reduction in the risk index (number of receptacles containing *Ae*. *albopictus* larvae per 100 households)^3^
[37]	2014/Reunion Island	n/r	0.39% of households	1024	n/r
[88]	2014/Reunion Island	Deltamethrin	n/r	162 (week 1), 55 (week 2), 65 (week 4), 49 (week 6)	Human antibody response to *Ae*. *albopictus* salivary proteins decreased over a 6 week period
[55]	2016/Reunion Island	Deltamethrin	n/r	n/r	Human Ab response to *Ae*. *albopictus* salivary proteins decreased^3^ Decreased house and Breteau indices^3^
Australasia					
[41]	2014/Australia	Pyrethroid	n/r	n/r	Decrease in Ae. albopictus^3^
[68]	2016/Australia	n/r	Cases 0.05%, controls 0.08%	102 (43/59)^4^	No difference in CHIKV infection rates between those using insecticide and those that don’t (p = 0.7)
**Larvicide**					
Europe					
[42]	2013/Spain	Diflubenzuron	16.4% (2008), 17.1% (2009), 6.1% (2010)	428 (2008), 245 (2009), 147 (2010)	n/r
Asia					
[85]	1975/Burma	Abate	n/r	n/r	Reduced house index^3^
[51]	2007/India	Abate	n/r	n/r	Reduced incidence of CHIKV cases^3^
[73]	2010/India	Abate	67.5% of households	1301	n/r
[89]	2011/Singapore	n/r	n/r	n/r	Median larval densities in clusters dropped from 380 in 2008 to 100 in 2009, (p = 0.011)
[46]	2011/India	Abate	CHIKV cases 0%, controls 60%	600 (150/450)	Greater use of insecticides among CHIKV negative controls than among cases (p<0.001)
[86]	2012/India	Temephos	n/r	n/r	Decreased number of suspected cases^3^
[87]	2015/India	Temephos	n/r	n/r	Decline in CHIKV cases 3 weeks following application^3^
Indian Ocean Islands					
[44]	2012/Reunion Island	Pyriproxyphen and Spinosad	n/r	n/r	Reduction in the risk index (number of recepticles containing *Ae*. *albopictus* larvae per 100 households).^3^
**Unspecified Use**					
Europe					
[48]	2010/Italy	n/r	n/r	325	No effect of pest control measures on risk of CHIKV infection OR 0.58 (95% CI 0.28–1.20, p = 0.14)
South America					
[39]	2014/Colombia	n/r	84% use chemical control	171	n/r
Indian Ocean Island					
[65]	2008/Reunion Island	n/r	n/r	n/r	Use of insecticides in the home did not decrease the number of CHIKV infected individuals (p = 0.41)

^1^ Cases/controls = number of individual who were positive for CHIKV/ negative for CHIKV in the study.

^2^ Indicates that the measure was not reported.

^3^ Extractable data was not provided in the article.

^4^ Individuals were cases if they were CHIKV or dengue positive.

The proportion of the population using adulticides was reported in 11 studies [33, 37, 38, 42, 46, 56, 63, 68, 70, 73, 74] and ranged from 0.39% [37] to 97% [46], Table 3. Outcomes of adulticide use were reported in 13 studies, Table 3. Decreases in the vector population were reported in eight studies as a reduction in a larval index [43, 44, 49, 55, 85], a decreased catch rate of adult mosquitoes [41, 62], and a decrease in median larval densities [81]. Five studies looked at rates of infection and found that there was a decrease in the number of chikungunya cases following adulticide fogging [86, 87], in two studies households not using adulticides had greater odds of contracting CHIKV, OR 1.2 (p<0.05) [36] and 3.22 (95%CI 2.27–4.55) [46]. The remaining study found no difference in infection rates between households using adulticides and those who did not (p = 0.7) [68]. A decrease in human antibody response (Ab) to *Ae*. *albopictus* salivary proteins was also reported in two studies, indicating a decrease in mosquito bites with adulticide use [55, 88].

The proportion of the population using larvicides was reported in three studies [42, 46, 73] and ranged from 0% [46] to 67.5% [73], Table 3. Outcomes of larvicide use were reported in seven studies, Table 3. Three of these studies reported a reduction in the vector population [44, 85, 89], and four reported a reduction in the number of chikungunya cases [46, 51, 86, 87].

Unspecified insecticide use was described in three studies, Table 3. One study reported that 84% of the study population was using chemical control [39]. The other two studies found that use of insecticides in the home did not decrease the number of CHIKV infected individuals [65] and there was no significant association between the use of pest control measures and CHIKV infection, OR 0.58 (95% CI 0.28–1.20) [48].

Although there was significant heterogeneity within each of the subgroups analysed, there were trends indicating that habitat removal was the most commonly employed mitigation measure. Random effects meta-analysis estimated the overall proportion of individuals that practice habitat removal is 65% (95% CI 55%-74%, I^2^ = 99.61%). Compared to the least used protective measure, physical barriers, which was estimated to be used by 25% (95% CI 17%-35%, I^2^ = 99.14%) of the study sample across studies. Insecticide was used by 30% (95% CI 17%-44%, I^2^ = 99.6%) of the sample population. The groups of studies examining habitat removal, physical barriers and insecticides had high heterogeneity between studies. Subgrouping by outbreak status did not explain a significant amount of heterogeneity, although there was a trend towards increased use of each mitigation measure during an outbreak compared to when no outbreak was occurring, Table 4.

**Table 4:** Meta-analysis results for the frequency of use of various mitigation and control measures for chikungunya virus, subgrouped by outbreak status.

Mitigation measure	Reference	Ongoing outbreak	n^1^	Pooled prevalence	95% CI (%)	*I*^*2*^
Room repellent	[34] [37] [38] [69–72]	Yes	11	41%	27–55	99.44%
	[63]	No	3	34%	12–60	94.48%
Personal repellent	[34] [37] [38] [64] [65] [68]	Yes	9	30%	13–49	99.62%
	[33] [63] [66] [67]	No	5	30%	12–52	98.77%
Unspecified repellent	[45] [58] [59] [68] [73]	Yes	5	69%	55–82	99.12%
	[42] [74] [75]	No	5	43%	31–57	95.16%
Physical barrier	[34] [35] [37] [38] [45] [58] [64] [69] [72]	Yes	10	29%	16–43	99.34%
	[42] [56] [63] [66] [67] [74] [75]	No	13	23%	12–37	98.96%
Insecticide use	[33] [44] [45]	Yes	10	29%	16–43	99.34%
	[32] [34] [46] [48]	No	13	23%	12–37	98.96%
Habitat removal	[33] [37] [38] [39] [53] [58] [61] [62]	Yes	12	72%	60–83	99.65%
	[33] [42] [56]	No	5	46%	29–64	99.09%

^1^The sample size may be larger than the number of references as there may be more than one data point extracted per study

### Biological mosquito control

Six studies mentioned the use of biological mosquito control [28, 39, 42, 61, 85, 90]. Of the six studies, one modeled the effects of biological mosquito control in a disease transmission model [28]. In 2013 a study in Spain investigated the introduction of *Bacillus thuringiensis* to water storage containers for larval control, but did not report a measure of effectiveness for the intervention [42]. A pilot study in 1975 in Burma added the larvivorous fish *Lebistes reticulatus* to water storage containers, however the water storage practices of the participants did not favour the survival of the fish for more than 24 hours [85]. In India (2011) the introduction of larvivorous fish to water storage containers and a complementary education campaign resulted in a significant reduction in *Aedes* larvae, OR 0.51 (p<0.001) [61]. In 2014 a study in Colombia reported that 73% (125/171) of the survey population was using biological mosquito control but did not provide any further details [39]. A study in Puerto Rico in 2016 reported on the use of autocidal gravid ovitraps to capture adult female *Ae*. *aegypti* and found that the proportion of chikungunya virus IgG antibody among participants from the two intervention communities was one half that of participants from control communities (risk ratio = 0.52, 95% CI 0.38–0.71) [90].

### Public education

Public education measures were described in 30 studies and covered topics such as how to avoid mosquito bites [30, 32, 36, 42, 57, 60, 80, 83, 86, 89, 91–94], how to recognise and remove vector breeding habitat [30, 32, 42, 44, 51, 53, 57, 59, 61, 78, 82, 83, 85, 86, 89, 91, 92, 94, 95], how to recognise chikungunya symptoms [53, 60, 61, 80, 92, 93], and general chikungunya information [53, 61, 73, 80, 83, 92, 96, 97]. Educational material was delivered through print media, either by newspaper or pamphlets [30–32, 44, 51, 57, 73, 80–82, 86, 95], in person to groups or individuals [36, 42, 51, 53, 60, 61, 83, 92–96], through social media [31], a website [93] or through television and radio [44, 80].

The impact of public health education campaigns was evaluated for the topics of vector habitat removal [32, 42, 44, 51, 57, 59, 85, 89, 93] and general chikungunya information [61, 92, 96, 97], Table 5. The public education campaigns that focused on recognising and removing vector breeding habitat resulted in a decrease in the number of breeding sites [92, 95], a reduction in the incidence of chikungunya cases [32, 51], a reduction in larval indices [44, 85, 89], prevention of local transmission following imported cases [57], and an increase in the number of individuals removing stagnant water [42]. For the public education campaigns that provided general chikungunya information, the impact of the campaign was measured as an increase in knowledge determined through the use of questionnaires [61, 92, 96, 97].

**Table 5:** Topic, delivery method, and effectiveness for public education campaigns aimed at reducing or preventing chikungunya transmission.

Ref	Publication year/location	Delivery method	Outcome
**Recognising and removing vector breeding habitat**			
North America			
[95]	2014/USA	In person and print media. Door to door and public spaces	22.6% reduction in container habitats in the communities being educated compared to a 32.3% increase in the sites not receiving education (p = 0.004)
[57]	2016/USA	Print media	No local transmission following nine imported cases
Europe			
[42]	2013/Spain	Delivered in person door to door	Increase over three years in the number of individuals who remove stagnant water on their property (chi squared p<0.05)
Asia			
[85]	1975/Burma	n/r^1^	Reduced House Index from 50% to 15%
[92]	2006/India	In person to school children who then replayed it to family members	Decrease in the number of mosquito breeding sites (Z = 7.82, p = 0)
[51]	2007/India	In person and print delivered door to door and in public spaces	Reduced incidence of CHIKV cases
[89]	2011/Singapore	n/r	Median larval densities in clusters dropped from 380 in 2008 to 100 in 2009
[32]	2013/India	Print media delivered door to door	Reduced incidence of CHIKV cases
Indian Ocean Islands			
[44]	2012/Reunion Island	High media coverage	Reduction in the risk index (number of receptacles containing *Ae*. *albopictus* larvae per 100 households)
**General chikungunya information**			
South America			
[96]	2014/Colombia	Delivered to medical students and professionals at a conference	Increase in the proportion of correct answers on a questionnaire after the intervention
Asia			
[92]	2006/India	Delivered in person to school children who then replayed it to family members	Change in knowledge scores on a questionnaire
[61]	2011/India	Delivered in person in public spaces	Increase in new knowledge determined through questionnaire administration
[97]	2012/India	Informational session delivered in the workplace	Increased knowledge regarding mosquitoes and control measures

^1^Indicates the measure was not reported

Other topics of public education included avoiding mosquito bites [30, 32, 36, 42, 53, 80, 83, 86, 89, 92, 94] and information on insecticide fogging [30], however quantification of the impact of the campaigns were not reported. One study [84] described mitigation measures implemented in various countries and did not provide any further details on the use or effectiveness of these measures.

### Control of blood products

Nine studies reported the control or treatment of blood and blood products in order to lessen the risk of transmission through blood transfusions. The most common action was a ban on donations or temporary deferral of blood donors during a defined risk of being viremic period, Table 6. During an outbreak in Italy in 2007, officials implemented a temporary ban on blood donation for individuals living in the area of the outbreak, donation deferral for those who had visited the area during the outbreak, and a quarantine period for blood donated by individuals who had visited the area prior to the outbreak [98]. The Netherlands implemented a donation deferral from travellers returning from Thailand in 2013 and estimated the risk of a viremic traveller donating blood to be very low at 0.068 travellers per year [99]. Martinique quarantined blood donations in 2014 [100], as did Thailand [101], Table 6. One study completed in Singapore in 2011 mentioned that mass screening occurred, but provided no further details [52]. One study developed a screening method capable of detecting asymptomatic donors [102].

**Table 6:** Blood and blood product control methods used to prevent transfusion transmission of chikungunya virus and estimated risk of transfusion related infection.

Ref	Publication year/location	Screening method	Donation deferral period	Length of quarantine	Risk of infection
**Donation ban**					
[98]	2008/Italy	Pre donation questionnaire	21 days	5 days	Highest weekly estimated risk of yielding one viremic unit from an asymptomatic viremic donor was 1:3801
**Donation deferral**					
[99]	2013/ Netherlands	n/r^1^	n/r	n/a^2^	Modeled number of potentially infected donors returning from Thailand during a chikungunya outbreak was 0.068 infected donors / year
**Blood product quarantine**					
[100]	2014/ Martinique	Serology, post donation reporting of febrile symptoms	n/a	72-hour post donation quarantine	n/a
[101]	2014/Thailand	Serology, pre-donation questionnaire, enhanced post donation report	n/a	7 days	n/a

^1^Indicates the measure was not reported

^2^Indicates the measure was not applicable to that study

In areas where an active outbreak of CHIKV is occurring the risk of a viremic individual donating blood is significantly higher. For example, during an outbreak in Italy [98] the risk of blood from an asymptomatic viremic donor was estimated to be 1 in 3801 (95% CI not reported) and in Thailand in 2009, the same risk was 1 in 2429 (0.04%, 95% CI 0.02%–0.06%) [101]. As a follow up to the Thailand study, the authors created a disease transmission model to estimate the mean number of transfusion transmitted CHIKV cases that would have occurred in the absence of blood safety implementation measures. The model indicated that screening measures implemented during the 2009 outbreak effectively reduced the risk of transfusion transmission [103].

Treatment of blood products to reduce the risk of transmission was evaluated in two studies. One study successfully used amotosalen photochemical treatment (PCT) or riboflavin pathogen reduction treatment (PRT) to significantly reduce the number of plaque forming units per millilitre [104]. Another study used Theraflex UV-platelet system for pathogen inactivation which resulted in a significant log reduction in post treatment titres [105].

### Quarantine of infected individuals

Quarantine and isolation of infected individuals was described in five studies. Of these five studies, three were disease transmission models that used quarantine to predict the effects of different mitigation measures [26, 28, 78]. Other outbreak responses involved the rapid detection and isolation of infectious individuals together with vector control measures [89, 106].

## Discussion

This SR aimed to identify and summarize all of the research on the use and effectiveness of individual and community level mitigation and control measures for CHIKV. The results indicate that a variety of mitigation and control methods for chikungunya have been used worldwide, that a great deal of variability exists in the frequency of use of these mitigation measures, and that there is a lack of consistency among studies when reporting outcomes. While this review was able to identify some possible trends in the frequency of use of mitigation measures, it has done more to highlight the current gaps in knowledge and/ or reporting that prevent summarization of how effective mitigation measures are at the prevention or control of chikungunya.

Removal of breeding habitat was used by the greatest proportion of respondents across studies, and was also the most commonly reported of the personal protective measures (33/55). This could possibly demonstrate the effectiveness of public education in altering behaviour, as habitat removal was the most commonly reported topic of the education campaigns. However, only five studies described both public education and personal protective measures [32, 42, 44, 53, 61] and only three measured the effectiveness of education in terms of reductions in vector breeding habitat [42, 92, 95], which limits the evidence on how effective public education campaigns may be at affecting behaviour change.

Public education campaigns that focused on general chikungunya knowledge used pre and post questionnaires in order to evaluate how effective the campaign was in improving knowledge, however, this study design cannot measure or make inference to behaviour change and long term adoption of mitigation measures [107]. Methods of measuring behaviour change and impact are needed to fully evaluate the impact of public education campaigns on vector habitat removal or the use of personal protective measures.

Adulticide and larvicide studies examined effectiveness of use inside and outside the home. Outcomes ranged from vector density measurements at different intervals post application to the risk of CHIKV infection in humans based upon data collected through primary research and surveillance. For most mosquito related outcomes authors reported a significant decrease in mosquito or larvae populations. Similarly, outcomes of human infection reported either a decline in CHIKV cases following insecticide application or a protective effect of using insecticides in the home for most studies where there was a high prevalence of exposure to CHIKV. Despite the demonstrated effectiveness of insecticides in controlling vector populations, there are concerns associated with its use, such as the development of insecticide resistance as well as the potential effects on environmental and human health [108]. There are also economic considerations since the greatest cost-benefit of insecticide application in public spaces is likely to be achieved in small to medium sized towns, whereas in larger urban centres the cost may overcome the public health benefits [109].

Quantitative data was available for only 60% of the studies examining the use and effectiveness of personal and community interventions for the prevention of CHIKV. Studies that reported a measure of the effectiveness of a mitigation strategy typically had a pre- and post-intervention evaluation where the outcome measure was taken at two or more time points prior to and after intervention implementation. There was a lack of consistency in the reported outcome measures across studies, this prohibited meta-analysis and direct comparisons of the effectiveness of interventions captured in this SR. The insecticide category is a good example of this heterogeneity, where several studies did not specify whether an adulticide or larvicide was used, and each study had a different outcome measurement including rates of CHIKV infection, vector density, and larval counts. Although each of these outcome measures can evaluate whether or not an insecticide had an impact, they cannot be summarized together to evaluate the consistency of insecticide impact across studies.

The frequency at which various mitigation strategies were used within a population was extracted from 33.7% of studies. These were usually point in time cross-sectional surveys that could not evaluate how effective the intervention was at decreasing the mosquito density or preventing cases of CHIKV. The analysis on the proportion of individuals using various interventions was highly heterogeneous and as a result, the findings in Table 4 should be interpreted with caution. There was not enough data to conduct meta-regression or explore subgroups beyond outbreak status in order to try and explain some of the heterogeneity. It is possible that location and date of study may account for some of the variation between subgroups as there may be different preferences for certain mitigation or control measures in different locations, at different times and by different populations [110].

Much of the literature described more than one mitigation measure, with particular overlap occurring between behavioural measures, insecticide use, and public education (Tables 2, 3 and 5). This overlap makes it difficult to determine whether the measured impact in the study was due to one particular intervention or the combination of interventions. Research on similar vector-borne diseases suggests that a combination of interventions is likely more effective than a single intervention on its own [110, 111]. Thus, future research should consider study designs that can evaluate single strategy vs. multifaceted interventions. Data on the potential impact of a variety of options will facilitate future development of the most effective community level mitigation strategies.

A lack of reporting on the effectiveness of interventions for emerging diseases has been noted elsewhere [112] and this SR resulted in similar findings. With the exception of one randomized control trial [95] (public education) and one case control study [46] (insecticide use), the rest of the studies were prevalence surveys or cross-sectional studies, which can only provide point in time data on the sampled population. The latter can investigate associations between outcome measures and risk factors such as the use of an intervention. However, cross-sectional surveys cannot provide chronological evidence that the intervention preceded the outcome (e.g. CHIKV infection) and thus limits interpretation of the association. Thus the data available from the studies identified in this SR is limited and highlights the need for controlled trials that may provide cause-effect evidence while controlling for confounding variables, and for improved consistency in the selection of outcome measures.

The use of personal protection and behavioural mitigation measures captured in this SR tended to be greater during times of CHIKV outbreaks, suggesting that there may need to be a greater emphasis on mitigation and control measures during non-outbreak periods in order to decrease the risk of an outbreak occurring or the magnitude of an outbreak by reducing the likelihood of local transmission [113]. This observation may be transferable to other mosquito-borne diseases as well, although this review only examined personal protective and behavioural mitigation measures during CHIKV outbreaks compared to periods when no outbreak was occurring. At an individual level, the motivation to practice protective behaviours is likely higher during an outbreak due to perceived risk of infection and normalization of protective behaviours by public education and media. The fact that habitat removal is the most prevalent form of mitigation, both during and not during an outbreak, could possibly be due to the fact that public education measures tend to focus on habitat and breeding site removal (Table 5). More research is needed to determine whether there is a relationship between the two, which would suggest that public education campaigns are successful in altering behaviour.

While there are some limitations to this SR, such as possible language bias due to the exclusion of articles in languages other than English, French, Spanish and Portuguese, or the possibility that research was missed by the scoping review search or not properly classified, every effort was made to minimize potential biases by developing the protocol and all tools used in the scoping review and this SR *a priori* and pre-testing them with the review team.

The results of this review could have implications for the mitigation of other mosquito borne viruses. *Aedes aegypti* and *Ae*. *albopictus* are also vectors for dengue and zika viruses, therefore any mitigation measure that is effective for CHIKV is likely to be effective in controlling local transmission of those viruses, and vice versa. As these viruses are undergoing rapid emergence in various parts of the world [114–116], identifying those measures that can prevent local transmission should be considered of public health importance.

## Conclusions

This SR summarized the knowledge on community level mitigation and control methods for CHIKV and highlighted current research gaps. There was a lack of research that assessed the effectiveness of various mitigation strategies. This SR focused on the prevention of CHIKV infection in humans, however many of the identified interventions are designed to control the vector or prevent mosquito bites (e.g nets, coils, insecticide), we acknowledge that there is other research measuring the effectiveness of these vector control and disease prevention strategies applied to other mosquito species and outcomes (e.g. mosquito bites, vector abundance) that were outside the scope of this SR. With respect to CHIKV research, future studies should focus on consistent reporting of outcomes and the standardisation of how outcomes are measured in order to be able to summarize the effectiveness of individual and complex interventions across studies. Being able to determine which mitigation and control strategies are the most effective will aid public health officials in designing effective education and vector control programs in order to reduce the risk of local transmission of CHIKV.

## Supporting information

S1 Appendix Protocol for systematic review on individual and community mitigation measures for prevention and control of chikungunya virus. (DOCX)

S2 Appendix Search strategy for scoping review of chikungunya virus. (DOCX)

S3 Appendix PRISMA flow diagram of articles through the scoping review process. (DOC)

S4 Appendix Relevant literature identified during the systematic review process. (DOCX)

S5 Appendix Data extracted from relevant articles during the systematic review process. (XLSX)

S6 Appendix PRISMA checklist for systematic review. (DOC)
