# Supplementary material for: A systematic review of individual and community mitigation measures for prevention and control of chikungunya virus
Source: PLoS One. 2019 Feb 27;14(2):e0212054. doi: 10.1371/journal.pone.0212054 (PMC6392276; doi:10.1371/journal.pone.0212054)
Supplement: S1 Appendix — (DOCX) [file pone.0212054.s001.docx]

# Title:

A systematic review of individual and community mitigation measures for prevention and control of chikungunya virus (CHIKV).

# Authors:

Catherine Hierlihy^1,2^, Lisa Waddell^2^, Judy Greig^2^, Ian Young^3^, Tricia Corrin^1,2^, Mariola Mascarenhas^2^

^1^Department of Population Medicine, University of Guelph

^2^Public Health Risk Sciences Division of the National Microbiology Laboratory at Guelph, Public Health Agency of Canada.

^3^School of Occupational and Public Health, Ryerson University

# Important dates:

Evidence published up to January, 2017

Protocol as of July, 2017

Contents

[Title: 1](#_Toc469847430)

[Authors: 1](#_Toc469847431)

[Important dates: 1](#_Toc469847432)

[Background 3](#_Toc469847433)

[Objectives 3](#_Toc469847434)

[Study Question 3](#_Toc469847435)

[Study Outputs 4](#_Toc469847436)

[Methods 4](#_Toc469847437)

[Review Team Expertise and Responsibilities 4](#_Toc469847438)

[Search Strategy 4](#_Toc469847439)

[Relevance Screening (RS) 4](#_Toc469847440)

[Inclusion/exclusion criteria 4](#_Toc469847441)

[Quality Assessment and Data Extraction (QA-DE) 5](#_Toc469847442)

[Review Management 5](#_Toc469847443)

[Data Analysis 5](#_Toc469847444)

[Appendix 1: QA-DE Form 6](#_Toc469847445)

# Background

Chikungunya virus (CHIKV) is a mosquito-borne virus that is transmitted to humans by mosquitoes from the *Aedes* genus, most commonly *Ae. aegypti* and *Ae. albopictus*. While CHIKV has been endemic in many parts of Africa and Asia for decades, it has recently re-emerged and spread to new areas including the pacific islands, South America, and the Caribbean where it was not previously recorded (Powers and Logue, 2011). Outbreaks on Réunion Island (2005-06) followed by Italy (2007), France (2010), the Caribbean islands (2013) and South America (2014) have made CHIKV a global public health issue, as the range of affected areas continues to increase and non-endemic countries are experiencing increases in travel-related CHIKV infections (Harter et al, 2014; Fernandez-Garcia et al, 2016). These outbreaks demonstrate that CHIKV can be imported to new areas where *Ae. albopictus* and *Ae. aegypti* are present*,* including a high proportion of the U.S. and Europe (Vega-Rua et al, 2013; Vega-Rua et al, 2014).

Although present in some of the same areas as dengue and malaria, CHIKV has historically received far less attention due to the self-limiting symptoms and a low risk of death. Symptoms are nonspecific and include febrile arthralgia, myalgia, headache, and rash which typically resolve within a few weeks (Pialoux et al, 2007), however, in a proportion of infected individuals the arthralgia is incapacitating and may lead to a chronic condition (Queyriaux et al, 2008). The 2005 outbreak on Réunion Island was well recorded and provides the basis for our understanding of the impact of the current CHIKV strain, including complications such as encephalopathy (Paquet et al, 2005), and in utero transmission of CHIKV (Ramful et al, 2007). The reported case fatality rate was approximately 1/1000 (Josseran et al, 2006).

There is no available vaccine or antiviral treatment for CHIKV. Therefore, prevention relies primarily on individual personal protective measures and community vector control measures. Recommendations by the Unites States Center for Disease Control and Prevention (CDC) include controlling mosquito breeding by removing stagnant water used as breeding sites, the use of insecticides and repellents, and wearing protective clothing (CDC 2006). However, it is unclear which strategy or combination of strategies is most effective in the prevention of CHIKV. An understanding of which mitigation measures are used and which are most effective will be useful for guiding future research and development of educational and vector control measures to assist in reducing the risk of local transmission of CHIKV.

A systematic review (SR) was conducted in order to summarize which individual and community level prevention and control strategies have been investigated and which are most effective in preventing or reducing transmission of CHIKV.

# Objectives

## Research Question

**What community and individual prevention and control strategies have been used in order to prevent or reduce transmission of chikungunya virus and how effective are they?**

This research aligns with PHRSD priorities, which include enhancing and guiding public health decision-making and policies by providing the authoritative analyses, recommendations and scientific collaborative services (using methods such as epidemiological studies and knowledge synthesis) to address the occurrence, trend and determinants of infectious disease in Canada with expert focus on the prevention of public health risks arising from the food chain, animals and the environment (LFZ, 2013).

## Definitions

**Chikungunya** is a virus which is transmitted by infected mosquitos and can cause illness in humans.

## Study Outputs

1. Primary systematic review of the evidence.
2. A repository and dataset of all relevant literature captured in this study.

# Methods

## Review Team Expertise and Responsibilities

| **Member** | **Organization** | **Project Role** |
| --- | --- | --- |
| Catherine Hierlihy | PHRS/NML | MPH Student –Project lead |
| Tricia Corrin | PHRS/NML | MPH Student |
| Lisa Waddell | PHRS /NML | Synthesis expertise |
| Judy Greig | PHRS /NML | Synthesis expertise |
| Mariola Mascarenhas | PHRS /NML | Synthesis expertise |
| Ian Young | Ryerson University | Synthesis expertise |

## Search Strategy

The published literature used for this SR is part of a scoping review of the global knowledge on chikungunya conducted by the PHRS team. Studies on mitigation and control methods for chikungunya were identified during the data characterization phase of this scoping review All relevant studies (n=91) identified during the scoping review will be considered for analysis. Only studies that identified humans as the host species will be included.

## Relevance Screening (RS)

This SR contains one relevance question to ensure the study is relevant to the scope of the research question. The question is based upon the inclusion/exclusion criteria and can be found in Appendix 1.

### Inclusion/exclusion criteria

Study must describe community mitigation and control methods used in order to prevent CHIKV infection.

## Quality Assessment and Data Extraction (QA-DE)

Relevant information from the studies with be extracted using the predesigned form in Appendix 1. Data will be extracted from each article by two independent reviewers and any disagreements between the reviewers will be resolved.

The data extracted from the studies will include; study design, risk of bias, type of mitigation measure(s), details of mitigation measure(s) implementation, and outcome data. The methodological quality of the studies will be evaluated using a quality assessment form available in Appendix 1. Previously designed critical appraisal tools for qualitative and quantitative studies were used to create the quality assessment form (GRADE, 2016). The quality assessment will be used to determine the level of confidence that can be placed in the results.

The QA-DE form in Appendix 1 was designed to be used for two different SRs simultaneously and therefore also contains questions on perceptions, attitudes, and/or knowledge of CHIKV. These questions do not apply to this SR

## Review Management

Data extraction and quality assessment will be conducted using the web-based systematic review software DistillerSR. This data will then be exported to Microsoft Excel for data cleaning and descriptive analysis.

## Data Analysis

All pertinent information regarding community level CHIKV prevention and control measures will be analysed descriptively and through meta-analysis where possible. Findings and recommendations, methods incorporated and their usefulness, and study limitations will also be captured. All evidence gathered will be summarised and discussed.

# Appendix 1: QA-DE Form

**What community and individual prevention and control strategies have been used in order to prevent or reduce transmission of chikungunya virus and how effective are they?**

**Note:** Remember to only extract information for the applicable question – **not all questions apply.** Be very specific about the data you extract AND only **extract primary information** (information collected by the author in the course of the experiment.)

| **Question** | **Options** | **Definitions/Additional notes** |
| --- | --- | --- |
| **Relevance Verification** | | |
| Which of the following does the paper describe? | - Social impacts, risk perceptions, attitudes and/or knowledge of CHIKV among the public and health professionals - Mitigation strategies to prevent CHIKV infections - Other, please specify: [text]   (Exclude, please submit) |  |
| **If exclusion criteria were selected above, submit the form before proceeding** | | |
| **General Questions** | | |
| What is the study design?  *(Check all that apply)* | - Observational study   - Cross-sectional   - Cohort   - Case-control   - Prevalence survey   - Surveillance or monitoring program   - Case study or case-series   - Outbreak investigation   - Sporadic cases investigation   - Longitudinal study   - Evaluation of diagnostic tests   - Other, please specify: _____ - Experimental study - Controlled trial - Challenge trial - Quasi experiment - Other, please specify:_____ - Qualitative study, please specify:________ - Mixed Methods, please specify: - Economic Model - Disease Transmission Model - Risk Assessment - Vector Mapping Model - Descriptive Study - Other, please specify: ___ | **Observational study** - Assignment of subjects into a treated group versus a control group is outside the control of the investigator   - **Cross-sectional -** Examines the relationship of a risk factor and outcome (disease) at a point in time on representative samples of the target population - **Cohort study -** is a study in which individuals with differing exposures to a suspected risk factor are observed through time for occurrence of an outcome - **Case-control study** - compares exposure to the risk factor in subjects who have an outcome (the 'cases') with subjects who do not have the outcome, but are otherwise similar (the 'controls') and drawn from the same sampling frame - **Prevalence survey -** Measurement of an outcome at a point in time but doesn’t measure or investigate potential predictors – include here routine monitoring or surveillance data collection - **Case or case-series -** a descriptive study of a single individual (case report) or small group (case series) - **Outbreak investigation -** Studies an outbreak retrospectively or while it is occurring. An outbreak is a sudden increase in the occurrence of CHIKV illness in a given area with linked cases - **Sporadic cases investigation -** Studies cases of CHIKV infection that are not linked in space and/or time - **Longitudinal study -** A research method in which data is gathered for the same subjects over a period of time - **Evaluation of Diagnostic Tests -**   One or more diagnostic test is  evaluated for sensitivity, specificity  against a gold standard, clinical  symptoms or another test    **Experimental study -** Each subject is assigned to a treated group or a control group before the start of the treatment   - **Control trial -** an experimental study in which people are allocated to intervention groups and evaluated for outcomes - **Challenge trial -** An experiment where subjects are artificially challenged or exposed to the disease agent and then allocated to the intervention groups for evaluation of the outcome - **Quasi-experiment -** An experiment in which subjects are not randomly assigned to groups. Often this is the method of choice in field trials where the samples of the outcome are taken from the same individuals before and after the experiment/intervention   **Qualitative study -** Aimed at understanding social phenomena, exploring issues, and answering questions of “why” and “how.” Please specify the design/methodology that is identified by the author, and if none is identified explicitly then indicate “not specified”  **Mixed methods -** Tackles a research question using different research methodologies. Please specify the research methodologies used  **Economic models -** use mathematical equations to describe how costs are affected by different inputs. The structure of the equations reflects the model builder’s attempt to describe reality  **Disease transmission models -** are mathematical models used to link the biological process of transmission and the emergent dynamics of infection at the population level  **Risk assessment -** is the determination of quantitative or qualitative value of risk related to a situation and a recognized threat (hazard). Quantitative risk assessment requires calculation of the magnitude of the potential loss and the probability that the loss will occur  **Vector mapping -** is collection of data on spread/range of vector population (may use GIS)  **Descriptive study -** involves simply a description of measures that were taken. E.g. officials removed standing water from residences and encouraged personal protection measures |
| Did the study include a control/comparison group? | - Yes - Concurrent comparison - Pre and post comparison - No | **Pre-post comparisons -** i.e. uncontrolled before-after studies |
| Was there a follow up period? | - Yes   □ What was the duration of the  study? [text]  □ At what points did follow up  occur? [text]   - No | **Follow Up:** Did the authors return after an extended period in order to monitor the effectiveness of the intervention  **No** - The study was conducted at a single point in time  Follow up e.g. the study duration was 1 year. Follow up points occurred at 3, 6, and 12 months |
| Were any intervention efficacy outcomes sufficiently reported for potential use in meta-analysis? | - Yes - No - N/A | If no data was collected, then N/A. If some data was collected but is insufficient for meta-analysis, No |
| **Quality Assessment (QA)** | | |
| **Risk of Bias (mandatory)** | | |
| What type of study was this? | - Qualitative (move to set 1 of QA) - Quantitative (move to set 2 of QA) | **Qualitative** – select this box if interviews and/or focus groups were conducted and the authors summarized the findings into themes/ and quoted participants  **Quantitative -** Most studies should have sampled a population and provided summaries of the outcomes of the experiment, survey etc. that was done. (Most studies will have a quantitative component) |
| *Qualitative RoB* | | |
| Was there a clear statement of the research purpose/aims? | - Yes - No | Consider the following to make a judgement:   - Clarity of focus, explicit purpose given, supported by prior research |
| Was the research design and data collection strategy clearly described and appropriate to address the research aims? | - Yes - No | Consider the following to make a judgement:   - Rationale provided for research design/data collection strategy (including setting) - Research design and data collection strategy were appropriate to address the research purpose/question |
| Was the sampling strategy clearly described and appropriate to address the research aims? | - Yes - No | Consider the following to make a judgement:   - Selection criteria detailed, and description provided for how sampling was undertaken - Justification for sampling strategy and selection of participants given |
| Was the method of analysis clearly described and appropriate to address the research aims? | - Yes - No | Consider the following to make a judgement:   - Approach made explicit (e.g. thematic analysis, grounded theory) and described in depth - Discussion of how coding systems/conceptual frameworks evolved - If thematic analysis conducted, is it clear how themes were derived? |
| Were the findings clearly described and supported by sufficient evidence? | - Yes - No | Consider the following to make a judgement:   - Did data provide sufficient depth, detail and richness? (e.g. illustrative quotes) - Context described and taken into account in interpretation/results - Approaches taken to ensure robustness (e.g. multiple analysts, triangulation, member checking/participant validation of results) |
| Was there evidence of researcher reflexivity? | - Yes - No | Consider the following to make a judgement:   - Discussion of relationship between the researchers and participants during data collection - Researchers’ potential role and influence on study critically examined and/or discussed - Evidence of how problems/complications met were dealt with |
| Were ethical issues taken into consideration? | - Yes - No | Consider the following to make a judgement:   - Study approved by ethics committee - Sufficient details provided on how the research was explained to participants and whether ethical standards were maintained - Documentation of how autonomy, consent, confidentiality, anonymity were managed - Documentation of any ethical dilemmas and how they were resolved |
| Was there evidence of study relevance and transferability? | - Yes - No | Consider the following to make a judgement:   - Discussion of contribution of study to existing/prior knowledge, practice, and/or policy - Areas for future research identified - Limitations/weaknesses of study clearly outlined - Discussion of whether or how the findings can be transferred to other populations or consideration of other ways the research may be used |
| *Quantitative RoB* | | |
| Was the allocation sequence adequately generated?  **Experiments | - Yes [text] - Unclear [text] - No [text] - N/A [text] | *****FOR 2 GROUPS*****  **Yes** - allocation sequence is described in sufficient detail. Individuals/samples are allocated to groups at the beginning  **Unclear** - they simply stated that it was “randomized” (formerly partial)    **No** - sample drawn without a formal process of random selection: judgment, convenience, purposive |
| Was the allocation sequence adequately concealed from the participants and the researcher?  **Experiments | - Yes [text] - Unclear [text] - No [text] - N/A [text] | **Yes** - concealment was sufficient and allocation was unlikely to be foreseen  **Unclear** – insufficient information provided to permit judgement  **No** – allocation sequence was not concealed |
| Was blinding appropriate? i.e. was knowledge of the status of the individual or sample adequately prevented during the study?  **Cohort | - Yes [text] - Unclear [text] - No [text] - N/A [text] | **Yes** - the authors state explicitly that participants were blinded  **Unclear** - insufficient information provided to permit judgement  **No** - knowledge of the intervention/ status of the individual or sample was not prevented during the study |
| Were the study participants (samples) selected randomly so the sample reflects outcomes and exposure in the population of interest?  **Cross-sectional | - Yes [text] - Unclear [text] - No [text] - N/A [text] | **Yes** – Random selection of the study participants or samples are stated and described  **Unclear** – insufficient information provided to permit judgement  **No** – Study participants were selected non-randomly or were not described |
| Were losses to follow-up (attrition) reported and comparable in both groups?  **Experiment & Cohort | - Yes [text] - Unclear [text] - No [text] - N/A [text] | **Yes –** If there are no losses to follow up, or if they are adequately reported. Distinction can be made in text box |
| Were exclusions from analysis reported?  **All | - Yes [text] - Unclear [text] - No [text] - N/A [text] | **N/A –** Descriptive Studies, or if there were no exclusions. Distinction can be made in text box. |
| Were confounders appropriately identified and accounted for?  **All | - Yes [text] - Unclear [text] - No [text] - N/A | **Yes** - All-important confounding factors were identified, accounted for by exclusion, matching or analysis  **Unclear -** insufficient information provided to permit judgement  **No** - Not stated  **N/A –** Descriptive Studies |
| Did the authors report all intended outcomes?  **All | - Yes [text] - Unclear [text] - No [text] | **Yes** - there is no evidence that outcomes were selectively reported (e.g. all relevant outcomes in the methods section are reported in the results section)  **Unclear -** insufficient information provided to permit judgement  **No** – outcomes are missing |
| If a questionnaire was used to measure outcomes, was it appropriately validated and reliably tested? | - Yes [text] - Unclear [text] - No [text] - N/A [text] | **Yes** – The questionnaire/focus group was appropriately validated and tested  **Unclear** – insufficient information to permit judgement  **No** – The questionnaire/focus group was not validated and/or tested |
| Was the study free of other problems that could put it at a high risk of bias?  **All | - Yes [text] - Unclear [text] - No [text] | **Yes -** I have no additional concerns about the design and/or conduct and reporting of this study  **Unclear** – insufficient information provided to permit judgement  **No -** the following are concerns I have that this study is at risk of bias (list with page#) |
| Overall, based on the GRADE questions please indicate the risk of bias for this study  **All | - Low RoB - Unclear RoB - High RoB | **Low RoB -** no biases were indicated in the assessment. Thus plausible bias is unlikely in all key domains (within this study)  **Unclear RoB -** there are plausible bias that raises doubt about the results as some key domains are “unclear (within this study)    **High RoB -** in one or more of the domains serious plausible bias was identified (within the study) |
| **Data Extraction (DE) - Social Impact/Knowledge & Perceptions** | | |
| Population/Demographics | | |
| How were the participants selected? | [text] |  |
| Did the authors state why the population was selected? | - Yes, please specify: [text] - No [text] |  |
| What is the sample size (n) of the study? | [text] |  |
| Was the response/participation rate captured? | - Yes, please specify : [text] - No [text] |  |
| Indicate the number of men and women in the study: | - Men [text] - Women [text] - Not stated |  |
| Did sex have a significant impact on any of the outcomes? | - Yes, please specify: [text] - No [text] - Not stated |  |
| Indicate the age range, median, mean and with its measure of variability (SE/95%CI/SEM etc) as provided in the paper: | - Age range [text] - Mean [text] - Median [text] - Not reported - Other, please specify: [text] |  |
| Was any additional demographic information captured in the study results? If so, please specify. | - Socioeconomic status [text] - Education level [text] - Work status [text] - Size of household [text] - Ethnicity [text] - Other, please specify: [text] | Capture all of the details provided |
| **Data Collection Form:**  **You will need a new data collection form to report each set of outcomes separately.** | | |
| What category does the result fall under? | - Perceptions about toxic or environmental effects of control/protective measures (e.g. DEET) - Perceptions about the severity of CHIK disease or vulnerabilities - Knowledge, perceptions, and attitudes on mitigation practices - Knowledge on CHIK disease - Knowledge on CHIKV-harbouring vectors/how CHIK is transmitted - Other, please specify: [text] |  |
| Please provide a description/interpretation of the result:  **Outcome measure* | [text] | E.g. Participants intention to use mosquito repellent, Do participants know what type of mosquito transmits CHIKV etc |
| What is the sample size (n) for this outcome? | [text] |  |
| What were the results? | - % or fraction [text] - OR [text] - SE [text] - CI [text] - P value [text] - Other [text] | If adjusted, note model and factors adjusted by |
| Was there any other information about the results provided? | [text] |  |

| **Data Extraction (DE) – Mitigation measures** | | |
| --- | --- | --- |
| What mitigation strategies were investigated? *(Check all that apply)* | □ Vaccination  □ Personal behavioural protective  measures, including repellents  applied to a person  □ Use of insecticides applied to the  environment/home/water (eg:  fogging, water treatments, coils)  □ Biologic control of mosquitoes  □ Public education to decrease risk of CHIK disease  □ Limiting use of/treating blood products  □ Other, please specify: [text] | **Behavioural** - protective measures include personal protection as well as physically removing potential breeding sites in the home (i.e. standing water in pots).  **Repellents** are products used to repel mosquitoes from a person or the area, and don’t necessarily have the express purpose of killing them.  **Insecticides** refer to chemical products used with the intent of killing mosquitoes (adults as well as eggs and larvae)  **Biologic control** - involves the use of other mosquito species, larvivorous fish or copepods, bacteria, sterile insects, etc  With respect to scope, we are not interested in the efficacy of large vector control campaigns, ONLY vector control practices that can be applied by an individual to themselves or their home. |
| Reason for implementation of mitigation measures: | □ Response to CHIKV outbreak  □ Response to other virus outbreak  □ General mosquito prevention  □ Other, please specify: [text] |  |
| What is the target audience of the intervention? *(Check all that apply)* | - General public - Physicians/healthcare workers - Adults - Children - Elderly - Other, please specify: [text] | ***Elderly =*** Seniors, >65 years old  ***Adults =*** 20-65 years old  ***Children =*** 19 years and under |
| *If vaccination selected* | | |
| Specify vaccine name, dose, frequency, and reported side effects: | - Name [text] - Vaccine details (target etc) [text] - Dose [text] - Frequency [text] - Reported side effects [text] |  |
| What species were vaccinated? | - Humans - Other [text] |  |
| If study is an animal trial, were study hosts challenged? | □ Yes  □ Days following vaccination: [text]  □ No | Measure of prevention of CHIKV transmission to host |
| Were host immune responses measured after intervention/challenge? | □ Yes  □ When were immune responses  Measured (give days, weeks,  Etc)  □ No  □ What was the length of inferred immunity if reported? [text] |  |
| *If Personal Behavioural Measures Selected* | | |
| Were insect repellents used? | - Yes   □ Name of repellent: [text]  □ Frequency of application: [text]   - No | Insect repellents for personal use |
| Did the intervention involve treating clothing with permethrin? | - Yes   □ Was the duration of  effectiveness measured? If so,  please specify: [text]   - No |  |
| Were mosquito/bed nets used? | - Yes   Was the net treated with an insecticide?: [text]   - Yes, please specify the   Insecticide: [text]   - No - Not reported - No |  |
| Was a dose and/or duration of intervention reported? | - Yes   □ Dose/frequency: [text]  □ Duration: [text]   - No | **Dose/frequency** – Quantity and frequency of the behaviour  **Duration** - How long was the behaviour repeated (e.g. days, weeks, months) |
| Was intervention effectiveness measured? | - Yes   □ What was the outcome? [text]  □ How was it measured? [text]   - No |  |
| *If Use of Insecticide Applied to Environment/Home/Water selected* | | |
| Where were insecticides applied (*Check all that apply)* | □ Standing water  □ Private dwellings  □ Public spaces  □ Other, please specify: [text] | With respect to scope, we are not interested in the efficacy of large vector control campaigns, ONLY vector control practices that can be applied by an individual to themselves or their home.  **Public spaces** - transit, roads, squares, government buildings open to the public |
| Was dose and/or frequency of application mentioned? | - Yes   □ Dose: [text]  □ Frequency: [text]   - No | **Dose -** Strength of insecticide applied  **Frequency -** How often insecticide was applied |
| Was intervention effectiveness measured? | - Yes   □ What was the outcome? [text]  □ How was it measured? [text]   - No |  |
| *If Biologic Control of Mosquitoes Selected* | | |
| At what lifestage was biologic control directed | - Egg - Larvae - Nymph - Adult |  |
| Was dose and/or frequency of intervention mentioned? | - Yes   □ Dose: [text]  □ Frequency: [text]   - No |  |
| Was intervention effectiveness measured? | - Yes   □ What was the outcome? [text]  □ How was it measured? [text]   - No |  |
| *If Public Education Selected* | | |
| How was the intervention delivered*? (Check all that apply)* | - In person   □ One on one  □ In a group   - Online/web based educational modules - Print media - TV - Radio - Multimedia - Website information - Social media - Other, please specify: [text] | **Print media** - e.g. newspapers, newsletters, brochures, posters  **Multimedia** – Any combination of print/text, audio, images, video, and/or interactive activities  **Social media** – Interactive exchange of information in virtual communities and metworks (eg: forums, Facebook, Twitter, YouTube) |
| What were the topics covered by the intervention? *(Check all that apply)* | - General CHIK information - How to avoid mosquito bites - Recognising and getting rid of mosquito breeding sites - How to recognise CHIK symptoms - Not reported - Other, please specify: [text] |  |
| Where was the intervention delivered *(Check all that apply)* | - Mailed to homes - Door to door homes - Public spaces - Hospitals/clinics/healthcare settings - Internet - Print media - Other, please specify: [text] | Public spaces: transit, roads, squares, parks, government buildings open to the public. |
| How often, and for how long, did the intervention take place? | - How often? [text] - How long? [text] - Not reported | **How often -** refers to how often the intervention was applied (e.g. number of sessions conducted)  **How long -** refers to how long the intervention was applied for.  NOTE: Where possible provide units of measurement (e.g. total hours, weeks, etc) |
| How was intervention effectiveness measured? | - Questionnaire - In person - Phone - Postal - Web based - Participant observation, specify details: [text] - Interviews - Other, please specify: [text] - Not reported/no follow up |  |
| How was intervention success determined? | - Test scores - Recognition of program messages - Changes in behaviour/practices   □ Self-reported  □ Observed   - Other, please specify: [text] - Not reported |  |
| Was the intervention successful? | - Yes, specify measure: [text] - No, specify measure: [text] - Not reported |  |
| *If Limiting use of/treating blood products selected* | | |
| Were blood donations screened for CHIKV? | - Yes   □ Serology  □ PCR  □ Pre-donation questionnaire  □ Other, please specify: [text]   - No |  |
| Were precautionary measures instituted for blood donations? | - Ban on blood donation from people living in affected area - Deferral period for people who visited the area   □ Length of deferral period? [text]   - Quarantine period for donated blood and/or blood products   □ Length of quarantine period? [text]   - Pathogen inactivation   □ Method: [text]   - Other, please specify: [text] |  |
| Do the authors attempt to predict risk of infection through transfusion? | - Yes   □ Risk of infection, with units [text]   - No | E.g. The weekly risk of yielding one  viremic unit from an asymptomatic viremic donor |
| *If Other Selected* | | |
| Was there a mitigation strategy that was not listed above? | - Describe the intervention: [text] - What outcome was measured?[text] - How was the outcome measured? [text] |  |

| **Data Collection Form:**  **You will need a new data collection form to report each set of outcomes separately.** |
| --- |

| Please provide a description/interpretation of the result:  **Outcome measure* | [text] | **E.g.** Community members were educated on the importance of removing standing water, resulting in a decrease in the number of mosquitoes |
| --- | --- | --- |
| What is the sample size for this outcome? | [text] |  |
| If the study failed to evaluate the impact of the intervention, but simply took a cross-section of a population at a point in time please indicate the outcomes captured | - % or fraction [text] - OR [text] - SE [text] - CI [text] - P value [text] - Other [text] |  |
| If the intervention was evaluated please fill in the results in the appropriate section below: | | |
| Difference in means (between intervention/  control groups): | Difference in means (value) [text]  N (total sample size) [text]  Common SD [text]  SE [text]  Variance [text]  CI interval [text]  P value [text]  T value [text]  Outcome units [text]  Was outcome adjusted for other variables? Please specify: [text] |  |
| Mean change from baseline (between pre-post values): | Specify group [text]  Difference in means (value) [text]  N (total sample size) [text]  Common SD [text]  SE [text]  Variance [text]  CI interval [text]  P value [text]  T value [text]  Outcome units [text]  Was outcome adjusted for other variables? Please specify: [text] |  |
| Dichotomous/  Ordinal Data: | Raw 2x2 table data:   - No. positive in group 1 [text] - No. negative in group 1[text] - No. positive in group 2 [text] - No. negative in group 2[text] - Define group 1 [text] - Define group 2 [text] - Specify “positive” [text] - Specify “negative” [text] - For matched studies, indicate external correlation [text] - If greater than two groups, specify data for other groups [text]   Computed effect size/measure of association:  Measure of association (value) [text]  Define what a positive or beneficial effect means versus a negative/harmful effect [text]  Specify measure (OR, RR, etc.) [text]  N in group 1 [text]  N in group 2 [text]  Define group 1 [text]  Define group 2 [text]  SE [text]  Variance [text]  Lower CI [text]  Higher CI [text]  Was outcome adjusted for other variables? Please specify: [text] | **Only answer based on how outcome data are REPORTED**  **Dichotomous -** Sufficient information includes:   - - Numerator ***and*** denominator, ***or***   - proportion + EITHER numerator or denominator ***or*** - Measure of association (e.g. odds ratio, relative risk) + EITHER a measure of variability (SE, CIs, variance) *or* an exact P-value   **Ordinal -** proportions reported for >2 ordinal/Likert scale categories (see required information for dichotomous data above). |
| Additional Comments? | [text] |  |

| **Final Questions**  ****ALL** |  |  |
| --- | --- | --- |
| What do the authors recommend be done with the research results? | [text] |  |
| Are there any important details that you believe were not extracted? | [text] |  |
