# Supplementary material for: A systematic review of individual and community mitigation measures for prevention and control of chikungunya virus
Source: PLoS One. 2019 Feb 27;14(2):e0212054. doi: 10.1371/journal.pone.0212054 (PMC6392276; doi:10.1371/journal.pone.0212054)
Supplement: S2 Appendix — (DOCX) [file pone.0212054.s002.docx]

### S2: Search Strategy for Scoping Review on Chikungunya Virus

(Obtained from M. Mascarenhas)

### Algorithms

### The initial search was conducted on May 27, 2015 using a pre-tested search algorithm: (Chikungunya OR CHIK OR CHIKV) OR (alphavirus AND mosquito* AND control). An updated search using the same electronic sources and algorithms was completed on January 6, 2017.

### Databases

### Scopus, PubMed/MEDLINE, Embase, CINAHL (Cumulative Index to Nursing & Allied Health), CAB, LILACS (South American), Agricola, and the COCHRANE Library for any relevant trials in the trial registry

### Grey Literature Sources and Procedures

A grey literature search of the pre-specified public health organization websites was undertaken between October 19 – 23, 2015 to identify any non-peer-reviewed studies or surveillance data that was not captured in the electronic search.

***Grey Literature searched:***

- World Health Organization (WHO) library (including SEARO, IMSEAR, IMEMR)
- The World Bank (*databank.worldbank.org/data/databases/infectious-diseases*
- Centers for Disease Control and Prevention (CDC) – Morbidity and Mortality Weekly Report (MMWR), FastStats – Infectious Disease ( [www.cdc.gov/nchs/faststats/infectious -disease.htm](http://www.cdc.gov/nchs/faststats/infectious%20-disease.htm), ArboNET
- Public Health Agency of Canada (PHAC)
- Public Health Ontario (PHO
- National Institutes of Health (NIH)
- Australia’s **National Notifiable Disease Surveillance System (NNDSS)**
- European Centre for Disease Prevention and Control (ECDC)
- **ENHanCEd Infectious Diseases (EID2 database); www.zoonosis.ac.uk/eid2**
- **Communicable Diseases Intelligence – Australian government’s Department of Health**
- Pan American Health Organization (PAHO)
- Eurosurveillance
- Global Health Database (<http://www.lshtm.ac.uk/library/resources/databases/info_globalhealthovid.html>)
- ProMED-mail
- Infochangeindia.org
- Asia Development Bank
- IndMED
- MedCarib
- Caribbean Public Health Agency (www.carpha.org)

###

### Search Verification

A snowball strategy for search verification was used. This included screening relevant paper references for potential relevance and if they were not already included in the scoping review database, they were added to the review for screening. The following were included in the snowball strategy:

- Hand searching MMWR and CDC website
- Screening reference lists (10 reviews)

Articles searched:

1. Mowatt, L., & Jackson, S.T. (2014). Chikungunya in the Caribbean: An Epidemic in the Making. Infect Dis Ther, 3:63-68.
2. Roques, P., & Gras, G. (2010). Chikungunya Fever: Focus on Peripheral Markers of Pathogenesis. The Journal of Infectious Diseases, 203:141-143.
3. Higgs, S., & Ziegler, S.A. (2010). A nonhuman primate model of Chikungunya disease. J Clin Invest, 120(3): 657-660.
4. Ogden, N.H., Lindsay, L.R., & Coulthart, M. (2015). Is there a risk of Chikungunya transmission in Canada? CCDR, 41-1.
5. Straetemans, M. (2008). Vector-related risk mapping of the introduction and establishment of Aedes Albopictus in Europe. Eurosurveillance, 13(1-3).
6. Weitzel, B.S., Rega, P.P., & Bork, C.E. (2012). Chikungunya virus: An emerging condition in the industrialized world. JAAPA, 25(1):E1-E5.
7. Parida, M.M., Santosh, S.R., Dash, P.K., & Rao, P.V.L. (2008). Rapid and real-time assays for detection and quantification of Chikungunya virus. Future Medicine, 3(2): 179-192.
8. Kar, P.K., Nagpal, B.N., Dua, V.K., Ghosh, S.K., Raghavendra, K., Bhatt, R.M., Anvikar, A., & Das, A. (2009). Molecular characterization of chikungunya virus from Andhra Pradesh, India. Indian J Med Res, 129: 335-337.
9. Cassadou, S., Boucau, S., Petit-Sinturel, M., Huc, P., Leparc-Goffart, I., & Ledrans, M. (2014). Emergence of chikungunya fever on the French side of Saint Martin island, October to December 2013. Euro Surveill. Available online: <http://www.eurosurveillance.org/ViewArticle.aspx?ArticleId=20752>
10. Kaur, P., & Chu, J.H. (2013). Chikungunya virus: an update on antiviral development and challenges. Drug Discovery Today, 18 (19/20):969-983.
