# Supplementary material for: A systematic review of individual and community mitigation measures for prevention and control of chikungunya virus
Source: PLoS One. 2019 Feb 27;14(2):e0212054. doi: 10.1371/journal.pone.0212054 (PMC6392276; doi:10.1371/journal.pone.0212054)
Supplement: S3 Appendix — (DOC) [file pone.0212054.s003.doc]

**S3. PRISMA flow diagram of articles through the scoping review processes**

**Screening**

**Included**

**Eligibility**

**Identification**

Records identified through database searching
(n = 17,406)

Additional records identified through other sources
(n = 0)

Records after duplicates removed
(n = 10,591)

Records screened
(n = 6815)

Records excluded
(n = 4895)

Full-text articles assessed for eligibility
(n = 91)

Full-text articles excluded
(n = 10)

9: research pertaining to vaccine development

1: duplicate study

Studies included in qualitative synthesis
(n = 81)

Studies included in quantitative synthesis (meta-analysis)
(n = 27)
