# Supplementary material for: A systematic review of individual and community mitigation measures for prevention and control of chikungunya virus
Source: PLoS One. 2019 Feb 27;14(2):e0212054. doi: 10.1371/journal.pone.0212054 (PMC6392276; doi:10.1371/journal.pone.0212054)
Supplement: S4 Appendix — (DOCX) [file pone.0212054.s004.docx]

# **S4: Relevant literature identified during SR process**

1. Ahmed S, Francis L, Ricketts RP, Christian T, Polson-Edwards K, Olowokure B. Chikungunya virus outbreak, Dominica, 2014. Emerg Infect Dis. 2015;21(5):909-11
2. Anand T, Kumar R, Saini V, Meena G, Ingle G. Knowledge and use of personal protective measures against mosquito borne diseases in a resettlement colony of Delhi. Ann Med Health Sci Res. 2014;4(2):227-32
3. Angelini P, Macini P, Finarelli AC, Po C, Venturelli C, Bellini R, et al. Chikungunya epidemic outbreak in Emilia-Romagna (Italy) during summer 2007. Parassitologia. 2008;50(1-2):97-8
4. Anish T, Vijayakumar K, Leela IA. Domestic and Environmental Factors of Chikungunya-affected Families in Thiruvananthapuram (Rural) District of Kerala, India. J Glob Infect Dis. 2011;3(1):32-6
5. Appassakij H, Promwong C, Rujirojindakul P, Wutthanarungsan R, Silpapojakul K. The risk of blood transfusion-associated Chikungunya fever during the 2009 epidemic in Songkhla Province, Thailand. Transfusion. 2014;54(8):1945-52
6. Appassakij H, Promwong C, Rujirojindakul P, Khuntikij P, Silpapojakul K. Risk of transfusion-transmitted chikungunya infection and efficacy of blood safety implementation measures: experience from the 2009 epidemic in Songkhla Province, Thailand. Transfusion. 2016;56(8):2100-7
7. Aswathy S, Dinesh S, Kurien B, Johnson AJ, Leelamoni K. A post-epidemic study on awareness of vector habits of chikungunya and vector indices in a rural area of Kerala. J Commun Dis. 2011;43(3):209-15
8. Balasubramaniam SM, Krishnakumar J, Stephen T, Gaur R, Appavoo N. Prevalence of Chikungunya in urban field practice area of a private medical college, Chennai. Indian J Communitty Med. 2011;36(2):124-7
9. Bedoya-Arias JE, Murillo-García DR, Bolaños-Muñoz E, Hurtado-Hurtado N, Ramírez-Jaramillo V, Granados-Álvarez S, et al. Healthcare students and workers' knowledge about epidemiology and symptoms of chikungunya fever in two cities of Colombia. J Infect Dev Ctries. 2015;9(3):330-32
10. Bloch D, Roth NM, Caraballo EV, Munoz-Jordan J, Hunsperger E, Rivera A, et al. Use of household cluster investigations to identify factors associated with chikungunya virus infection and frequency of case reporting in Puerto Rico. PLoS Negl Trop Dis. 2016;10(10):doi:10.1371/journal.pntd.0005075
11. Boyer S, Foray C, DeHecq JS. Spatial and temporal heterogeneities of Aedes albopictus density in La Reunion Island: rise and weakness of entomological indices. PLoS One. 2014;9(3): e91170
12. Campo Carey AR, Benavides Ocampo M , Martinez Duran ME, Caro Nunez OA, Gomez Amaya JA, Nieto Sanchez DL, et al. [Chikungunya outbreak in the municipality of Mahates, Bolivar, 2014]. Informe Quincenal - Epidemiologico Nacional. 2014;19(21);342-67 Spanish
13. Chebabi AG, Roiz D, Guitart R, Quitana S, Gimenez N. Control of the Asian Tiger mosquito (*Aedes albopictus*) in a firmly established area in Spain: risk factors and people's involvement. Trans R Soc Trop Med Hyg. 2013;107:706-15
14. Cherry CC, Beer KD, Fulton C, Wong D, Buttke D, Staples JE, et al. Knowledge and use of prevention measures for chikungunya virus among visitors - Virgin Islands National Park, 2015. Travel Med Infect Dis. 2016;14(5):475-80.
15. Chiu CY, Bres V, Yu G, Krysztof D, Naccache SN, Lee D, et al. Genomic assays for identification of chikungunya virus in blood donors, Puerto Rico, 2014. Emerg Infect Dis. 2015;21(8):1409-13
16. Delisle E, Rousseau C, Broche B, Leparc-Goffart I, L’Ambert G, Cochet A, et al. Chikungunya outbreak in Montpellier, France, September to October 2014. Euro Surveill. 2015;20(17): pii: 21108
17. D'Ortenzio E, Grandadam M, Balleydier E, Jaffar-Bandjee MC, Michault A, Brottet E, et al. A226V strains of Chikungunya virus, Reunion Island, 2010. Emerg Infect Dis. 2011;17(2):309-11
18. Doucoure S, Mouchet F, Cornelie S, Drame PM, D’Ortenzio E, DeHecq JS, et al. Human antibody response to Aedes albopictus salivary proteins: a potential biomarker to evaluate the efficacy of vector control in an area of Chikungunya and Dengue Virus transmission. Biomed Res Int. 2014;doi:10.1155/2014/746509
19. Dumont Y, Chiroleu F. Vector control for the Chikungunya disease. Math Biosci Eng. 2010;7(2):313-45
20. Faddy HM, Fryk JJ, Prow NA, Watterson D, Young PR, Hall RA, et al. Inactivation of dengue, chikungunya, and Ross River viruses in platelet concentrates after treatment with ultraviolet C light. Transfusion. 2016;56(6 Pt 2):1548-55
21. Flahault A, Aumont G, Boisson V, de Lamballerie X, Favier F, Fontenille D, et al. An interdisciplinary approach to controlling chikungunya outbreaks on French islands in the south-west Indian Ocean. Med Trop. 2011;72:66-71
22. Fritzell C, Raude J, Adde A, Dusfour I, Quenel P, Flamand C. Knowledge, Attitude and Practices of Vector-Borne Disease Prevention during the Emergence of a New Arbovirus: Implications for the Control of Chikungunya Virus in French Guiana. PLoS Negl Trop Dis. 2016;10(11):e0005081
23. Gabor JJ, Schwarz NG, Esen M, Kremsner PG, Grobusch MP. Dengue and chikungunya seroprevalence in Gabonese infants prior to major outbreaks in 2007 and 2010: A sero-epidemiological study. Travel Med Infect Dis. 2016;14(1):26-31
24. Gallian P, de Lamballerie X, Salez N, Piorkowski G, Richard P, Paturel L, et al. Prospective detection of chikungunya virus in blood donors, Caribbean 2014. Blood. 2014;123(23):3679-81
25. Genderen FT, Krishnadath I, Sno R, Grunberg MG, Zijlmans W, Adhin MR. First chikungunya outbreak in Suriname; clinical and epidemiological features. PLoS Negl Trop Dis. 2016;10(4):e0004625
26. Ghosh SK, Chakaravarthy P, Panch SR, Krishnappa P, Tiwari S, Ojha VP, et al. Comparative efficacy of two poeciliid fish in indoor cement tanks against chikungunya vector *Aedes aegypti* in villages in Karnataka, India. BMC Public Health. 2011;doi:10.1186/1471-2458-11-599
27. Goddard J, Varnado WC, Hand S, Meyer F. Chikungunya in Mississippi: The Health Department response to imported cases. J Miss State Med Assoc. 2016;57(5):138-41
28. Goorah S, Dewkurun MK, Ramchurn SK. Assessing the sustainability of individual behavior change against mosquitoes after the outbreak of vector-borne disease in Mauritius: a case study. Internet J of Medical Update. 2013;8(1):9-16
29. Gould LH, Osman MS, Farnon EC, Griffith KS, Godsey MS, Karch S, et al. An outbreak of yellow fever with concurrent chikungunya virus transmission in South Kordofan, Sudan, 2005. Trans R Soc Trop Med Hyg. 2008;102(12):1247-54
30. Grandadam M, Caro V, Plumet S, Thiberge JM, Souares Y, Failloux AB, et al. Chikungunya virus, southeastern France. Emerg Infect Dis. 2011;17(5):910-3
31. Healy K, Hamilton G, Crepeau T, Healy S, Unlu I, Farajollahi A, et al. Integrating the public in mosquito management: active education by community peers can lead to significant reduction in peridomestic container mosquito habitats. PLoS One. 2014;9(9):e108504
32. Ho K, Ang LW, Tan BH, Tang CS, Ooi PL, James L, et al. Epidemiology and control of chikungunya fever in Singapore. J Infect. 2011;62(4):263-70
33. Impoinvil DE, Ahmad S, Troyo A, Keating J, Githeko AK, Mbogo CM, et al. Comparison of mosquito control programs in seven urban sites in Africa, the Middle East, and the Americas. Health Policy. 2007;83(2):196-212
34. Jain R, Acharya AS, Khandekar J, Jais M. Entomo-epidemiological investigations of chikungunya outbreak in Delhi, India. Ann Trop Med Public Health. 2013;6:297-300
35. Jain S, Kadri S, Venkatesh S, Lal S, Katyal R. Epidemiological investigation of an outbreak of chikungunya in hyderabad and nalgonda districts of andhra pradesh, India. Int J Health Sci. 2007;1(2):303-8
36. Knope KE, Doggett SL, Kurucz N, Feldman R, Johansen CA, Nicholson J, et al. Arboviral diseases and malaria in Australia, 2011-12: annual report of the national arbovirus and malaria advisory committee. Commun Dis Intell Q Rep. 2014;38(2):E122-42.
37. Kuan G, Ramirez S, Gresh L, Ojeda S, Melendez M, Sanchez N, et al. Seroprevalence of anti-chikungunya virus antibodies in children and adults in Managua, Nicaragua, after the first chikungunya epidemic, 2014-2015. PLoS Negl Trop Dis. 2016;10(6):e0004773
38. Lalani T, Yun H, Tribble D, Ganesan A, Kunz A, Fairchok M, et al. A comparison of compliance rates with anti-vectorial protective measures during travel to regions with dengue or chikungunya activity, and regions endemic for Plasmodium falciparum malaria. J Travel Med. 2016;23(5): doi:10.1093/jtm/taw043
39. Leo YS, Chow ALP, LiKiang T, Lye DC, Li L, Ng LC. Chikungunya outbreak, Singapore, 2008. Emerg Infect Dis. 2009;15(5):836-7
40. Lieshout-Krikke RW, Zaaijer HL, Prinsze FJ. The yield of temporary exclusion of blood donors, exposed to emerging infections abroad. Vox Sang. 2013;104(1):12-18
41. Liew C,Yung CF. First detection of chikungunya infection and transmission in Brunei Darussalam. Singpore Med J. 2012;53(4):e66-8
42. Liu X, Stechlinski P. Application of control strategies to a seasonal model of chikungunya disease. Appl Math Model. 2015;39(12):3194-3220
43. Liumbruno GM, Calteri D, Petropulacos K, Mattivi A, Po C, Macini P, et al. The Chikungunya epidemic in Italy and its repercussion on the blood system. Blood Transfus. 2008;6(4):199-210
44. Lorenzi OD, Major C, Acevedo V, Perez-Padilla J, Rivera A, Biggerstaff BJ, et al. Reduced incidence of chikungunya virus infection in communities with ongoing Aedes aegypti mosquito trap intervention studies - Salinas and Guayama, Puerto Rico, November 2015-February 2016. MMWR Morb Mortal Wkly Rep. 2016;65(18):479-80
45. Majra JP, Acharya D. Impact of knowledge and practices on prevention of chikungunya in an epidemic area in India. Ann Trop Med Public Health. 2011;4(1):3-6
46. Mecoli M, De Angelis V, Brailsford SC. Using system dynamics to evaluate control strategies for mosquito-borne diseases spread by human travel. Comput Oper Res. 2013;40(9):2219-28
47. Mehta D, Solanki H, Patel P, Umat P, Chauhan R, Shukla S, et al. A study on knowledge, attitude & practice regarding mosquito borne diseases in an urban area of Bhavnagar. Indian J Prev Soc Med. 2015;6(2):29-32
48. Millman AJ, Esposito DH, Biggs HM, Decenteceo M, Klevos A, Hunsperger E, et al. Chikungunya and dengue virus infections among United States community service volunteers returning from the Dominican Republic, 2014. Am J Trop Med Hyg. 2016;94(6):1336-41
49. Moro ML, Gagliotti C, Silvi G, Angelini R, Sambri V, Rezza G, et al. Chikungunya virus in North-eastern Italy: a seroprevalence survey. Am J Trop Med Hyg. 2010;82(3):508-11
50. Moulay D, Aziz-Alaoui MA, Kwon HD(2012). Optimal control of chikungunya disease: Larvae reduction, treatment and prevention. Math Biosci Eng. 2012;9(2):369-92
51. Ndeffo-Mbah ML, Durham DP, Skrip LA, Nsoesie EO, Brownstein JS, Fish D, et al. Evaluating the effectiveness of localized control strategies to curtail chikungunya. Sci Rep. 2016;doi: 10.1038/srep23997
52. Ndille EE, Doucoure S, Poinsignon A, Mouchet F, Cornelie S, D'Ortenzio E, et al. Human IgG Antibody Response to Aedes Nterm-34kDa Salivary Peptide, an Epidemiological Tool to Assess Vector Control in Chikungunya and Dengue Transmission Area. PLoS Negl Trop Dis. 2016;10(12):e0005109
53. Nhan TX, Claverie A, Roche C, Teissier A, Colleuil M, Baudet JM, et al. Chikungunya virus imported into French Polynesia, 2014. Emerg Infec Dis. 2014;20(10):1773-74
54. Patil SS, Patil SR, Durgawale PM, Patil AG. A study of the outbreak of Chikungunya fever*.* J Clin Diagn Res. 2013;7(6):1059-62
55. Poletti P, Messeri G, Ajelli M, Vallorani R, Rizzo C, Merler S. Transmission potential of Chikungunya virus and control measures: the case of Italy. PLoS One. 2011;6(5): doi:10.1371/journal.pone.0018860
56. Prajapati A, Parikh S, Fancy M, Bala DV. Impact of educational intervention regarding mosquito borne diseases and their control measures among the link workers of urban health centers (UHCs) of Ahmedabad city. Natl J Community Med. 2012;3(2):178-82
57. Puwar T, Sheth JK, Kohli V, Yadav R. Prevalence of chikungunya in the city of Ahmedabad, India, during the 2006 outbreak: A community based study. Dengue Bull. 2010;34:40-5
58. Qiaoli Z, Jianfeng H, De W, Zijun W, Xinguang Z, Haojie Z, et al. Maiden outbreak of Chikungunya in Dongguan City, Guangdong Province, China: epidemiological characteristics. PLoS One. 2012;7(8): e42830
59. Raude J, Setbon M. The role of environmental and individual factors in the social epidemiology of chikungunya disease on Mayotte Island. Health Place. 2009;15(3):659-69
60. Raude J, Chinfatt K, Huang P, Betansedi CO, Katumba K, Vernazza N, et al. Public perceptions and behaviours related to the risk of infection with Aedes mosquito-borne diseases: a cross-sectional study in Southeastern France. BMJ Open. 2012;2(6):doi: 10.1136/bmjopen-2012-002094
61. Salje H, Lessler J, Paul KK, Azman AS, Rahman MW, Rahman M, et al. How social structures, space, and behaviors shape the spread of infectious diseases using chikungunya as a case study. Proc Natl Acad Sci USA. 2016;113(47):13420-25
62. Selvavinayagam TS. Chikungunya fever outbreak in Vellore, South India. Indian J Communitty Med. 2007;32(4):286-7
63. Setbon M, Raude J, Pottratz D. Chickungunya on Reunion Island: social, environmental and behavioural factors in an epidemic context. Population. 2008;63(3):491-519
64. Seyler T, Sakdapolrak P, Prasad SS, Dhanraj R. A chikungunya outbreak in the metropolis of Chennai, India, 2006. J Environ Health. 2012;74(6):8-13
65. Sorge F, Tamburro M, de Pertat T. [Use and effects of insect repellents in infants in Reunion Island (France) during the 2005-2006 chikungunya epidemic: the 2009 INR study]. Bull Epidemiol Hebd (Paris). 2011;6:54-9. French
66. Soulaphy C, Souliphone P, Phanthavong K, Phonekeo D, Phimmasine S, Khamphaphongphane B, et al. Emergence of chikungunya in Moonlapamok and Khong districts, Champassak Province, the Lao People's Democratic Republic, May to September 2012. Western Pac Surveill Response J. 2013;4(1):46-50
67. Staikowsky F, le Roux K, Schuffenecker I, Laurent P, Grivard P, Develay A, et al. Retrospective survey of Chikungunya disease in Reunion Island hospital staff. Epidemiol Infect. 2008;136(2):196-206
68. Surendran SN, Kannathasan S, Kajatheepan A, Jude PJ. Chikungunya-type fever outbreak: some aspects related to this new epidemic in Jaffna district, northern Sri Lanka. Trop Med and Health. 2007;35(3):249-52
69. Tan LK, Lam S, Low SL, Tan FH, Ng LC, Teo D. Evaluation of pathogen reduction systems to inactivate dengue and chikungunya viruses in apheresis platelets suspended in plasma. Adv Infect Dis. 2013;3:1-9
70. Tenglikar PV, Hussain M, Nigudgi SR, Ghooli S. Knowledge and practices regarding mosquito borne disease among people of an urban area in Kalaburgi, Karnataka. Ntl J Community Med. 2016;7(3):223-25
71. Thaung U, C. Ming K, Thein M. Dengue haemorrhagic fever in Burma. Southeast Asian J Trop Med Public Health. 1975;6(4):580-91
72. Thuilliez J, Bellia C, Dehecq JS, Reilhes O. Household-level expenditure on protective measures against mosquitoes on the island of La Réunion, France. PLoS Negl Trop Dis. 2014;8(1):e2609
73. Tsiodras S, Pervanidou D, Papadopoulou E, Kavatha D, Baka A, Koliopoulos G, et al. Imported chikungunya fever case in Greece in June 2014 and public health response. Pathog Glob Health. 2016;110(2):68-73
74. Uc-Puc V,Herrera-Bojorquez J, Carmona-Carballo C, Che-Mendoza A, Medina-Barreiro A, Chable-Santos J, et al. [Effectiveness of commercial repellents against Aedes aegypti (L.) in Yucatan, México]. Salud Publica Mex. 2016;58(4):472-5. Spanish
75. Uthappa CK, Allam RR, Gunti D, Nalini C, Udaragudi PR, Tadi GP, et al. Chikungunya outbreak in Atmakur village, Medak district, Telangana State, India. Indian J Med Res. 2015;142(Suppl 1): S108–S110
76. Vaidya V, Sawant S. A KAP study in Pune City involving school children as a strategy for effective vector control in chikungunya. Indian J Public Health and Dev. 2013;4(4):438-43
77. Van Bortel W, Dorleans F, Rosine J, Blateau A, Rousset D, Matheus S, et al. Chikungunya outbreak in the Caribbean region, December 2013 to March 2014, and the significance for Europe. Euro Surveill. 2014;19(13):pii=20759
78. Vijayakumar K, Anish TS, Streekala KN, Ramachandran R, Philip RR. Environmental factors of households in five districts of Kerala affected by the epidemic of chikungunya fever in 2007. Natl Med J India. 2010;23(2):82-4
79. Vilain P, Larrieu S, Renault P, Baville M, Filleul L. How to explain the re-emergence of chikungunya infection in Reunion Island in 2010? Acta Trop. 2012;123(2):85-90
80. Yoosuf AA, Shiham I, Mohamed AJ, Ali G, Luna JM, Pandav R, et al. First report of chikungunya from the Maldives. Trans R Soc Trop Med Hyg. 2009;103(2):192-6
81. Yoshikawa MJ, Tang CS, Nishibuchi M. Incidence of chikungunya fever in Singapore: implications of public health measures and transnational movements of people. Trop Med Health. 2010;38(1):39-45
