# Supplementary material for: A systematic review of individual and community mitigation measures for prevention and control of chikungunya virus
Source: PLoS One. 2019 Feb 27;14(2):e0212054. doi: 10.1371/journal.pone.0212054 (PMC6392276; doi:10.1371/journal.pone.0212054)
Supplement: S6 Appendix — (DOC) [file pone.0212054.s006.doc]

| **Section/topic** | **#** | **Checklist item** | **Reported on page #** |
| --- | --- | --- | --- |
| **TITLE** | | |  |
| Title | 1 | Identify the report as a systematic review, meta-analysis, or both.  A systematic review (SR) and meta-analysis was conducted in order to summarize which individual and community level prevention and control strategies have been investigated and which are most effective to prevent or reduce transmission of CHIKV. | 4 |
| **ABSTRACT** | | |  |
| Structured summary | 2 | Provide a structured summary including, as applicable: background; objectives; data sources; study eligibility criteria, participants, and interventions; study appraisal and synthesis methods; results; limitations; conclusions and implications of key findings; systematic review registration number. | 2 |
| **INTRODUCTION** | | |  |
| Rationale | 3 | Describe the rationale for the review in the context of what is already known. |  |
| Objectives | 4 | Provide an explicit statement of questions being addressed with reference to participants, interventions, comparisons, outcomes, and study design (PICOS).  The research question for this review is “what community level prevention and control strategies have been investigated and which have been the most effective at reducing transmission of CHIKV”. | 4 |
| **METHODS** | | |  |
| Protocol and registration | 5 | Indicate if a review protocol exists, if and where it can be accessed (e.g., Web address), and, if available, provide registration information including registration number.  The protocol is available in the supplementary material (S1 Appendix). | 5 |
| Eligibility criteria | 6 | Specify study characteristics (e.g., PICOS, length of follow-up) and report characteristics (e.g., years considered, language, publication status) used as criteria for eligibility, giving rationale.  The scoping review search was conducted to identify all primary research related to chikungunya in English, French, Spanish, or Portuguese. | 5 |
| Information sources | 7 | Describe all information sources (e.g., databases with dates of coverage, contact with study authors to identify additional studies) in the search and date last searched.  The scoping review search was conducted to identify all primary research related to chikungunya in English, French, Spanish, or Portuguese in seven databases: Scopus, PubMed, CINAHL, CAB, LILACS, Agricola and Cochrane. The search was conducted on May 27, 2015 and updated January 6, 2017 | 5 |
| Search | 8 | Present full electronic search strategy for at least one database, including any limits used, such that it could be repeated.  the search algorithm: (Chikungunya OR CHIK OR CHIKV) OR (alphavirus AND mosquito* AND control). | 5 |
| Study selection | 9 | State the process for selecting studies (i.e., screening, eligibility, included in systematic review, and, if applicable, included in the meta-analysis).  All studies on any aspect of chikungunya and its vectors were included and characterized by topic. All studies characterized as examining mitigation measures at the individual or community level to prevent/control CHIKV (n=91) were considered for inclusion in the SR | 5 |
| Data collection process | 10 | Describe method of data extraction from reports (e.g., piloted forms, independently, in duplicate) and any processes for obtaining and confirming data from investigators.  pretested tools (screening form, risk of bias tool, and the data extraction form). Available in Appendix S1.  All stages of the SR, relevance screening, risk of bias assessment and data extraction were completed by two independent reviewers for each study and conflicts between reviewers were resolved by consensus. | 5, 6 |
| Data items | 11 | List and define all variables for which data were sought (e.g., PICOS, funding sources) and any assumptions and simplifications made.  The data extraction form captured characteristics such as type and details of the intervention and data on all relevant outcomes. | 6 |
| Risk of bias in individual studies | 12 | Describe methods used for assessing risk of bias of individual studies (including specification of whether this was done at the study or outcome level), and how this information is to be used in any data synthesis.  The risk of bias assessment form was adapted from the tools endorsed by the Cochrane Collaboration and aimed to determine the internal validity of each study. Each study was rated as having a low, high, or unclear risk of bias based on 10 criteria that appraised the study design, reporting of methodology and data exclusions. | 6 |
| Summary measures | 13 | State the principal summary measures (e.g., risk ratio, difference in means).  proportion of the population using an intervention | 7 |
| Synthesis of results | 14 | Describe the methods of handling data and combining results of studies, if done, including measures of consistency (e.g., I^2^) for each meta-analysis.  All stages of the scoping review and SR were conducted using the web-based management software DistillerSR (DistillerSR, Evidence Partners, Ottawa, Canada) to facilitate reviewing. Extracted data were exported to MS excel (Microsoft Corporation, Redmond, WA, USA) for data cleaning and descriptive summary.  Heterogeneity was measured using *I*^2^, which describes the proportion of total variation in study estimates that is due to heterogeneity, and it was considered high if *I*^2^>50%.  If the prevalence of a particular mitigation measure was reported more than once in the same study, such as during different years, they were treated as separate studies for meta-analysis. | 6, 7 |

Page 1 of 2

| **Section/topic** | **#** | **Checklist item** | **Reported on page #** |
| --- | --- | --- | --- |
| Risk of bias across studies | 15 | Specify any assessment of risk of bias that may affect the cumulative evidence (e.g., publication bias, selective reporting within studies).  Each study was rated as having a low, high, or unclear risk of bias based on 10 criteria that appraised the study design, reporting of methodology and data exclusions. S1 Appendix | 6 |
| Additional analyses | 16 | Describe methods of additional analyses (e.g., sensitivity or subgroup analyses, meta-regression), if done, indicating which were pre-specified.  Sub-group analysis was conducted to determine whether any of the heterogeneity between studies could be explained. The proportion of the population employing various control measures were grouped by the control method used and included the use of personal repellent, room repellents, unspecified repellent use, physical barriers, habitat removal, insecticide use, and mosquito avoidance. | 7 |
| **RESULTS** | | |  |
| Study selection | 17 | Give numbers of studies screened, assessed for eligibility, and included in the review, with reasons for exclusions at each stage, ideally with a flow diagram.  There were 1920 articles characterized in the scoping review, 91 of which were identified as evaluating individual or community mitigation measures against CHIKV. Eighty-one of these studies were relevant to this SR review question, Figure 1. Of the ten excluded articles, one was excluded as a duplicate study and nine were on vaccine development for humans. PRISMA diagram included. | 7 |
| Study characteristics | 18 | For each study, present characteristics for which data were extracted (e.g., study size, PICOS, follow-up period) and provide the citations.  Table 1, list of references in Appendix S4 | 9 |
| Risk of bias within studies | 19 | Present data on risk of bias of each study and, if available, any outcome level assessment (see item 12).  Based on the risk of bias evaluation, the majority of studies scored low (51.9%) or unclear risk of bias (40.7%), whereas 8.6% were considered to have a high risk of bias, Table 1. | 8 |
| Results of individual studies | 20 | For all outcomes considered (benefits or harms), present, for each study: (a) simple summary data for each intervention group (b) effect estimates and confidence intervals, ideally with a forest plot.  Tables 2, 3, and 5 | 10, 19, 25 |
| Synthesis of results | 21 | Present results of each meta-analysis done, including confidence intervals and measures of consistency. | 23 |
| Risk of bias across studies | 22 | Present results of any assessment of risk of bias across studies (see Item 15).  Based on the risk of bias evaluation, the majority of studies scored low (51.9%) or unclear risk of bias (40.7%), whereas 8.6% were considered to have a high risk of bias, Table 1. | 8 |
| Additional analysis | 23 | Give results of additional analyses, if done (e.g., sensitivity or subgroup analyses, meta-regression [see Item 16]).  Table 4 | 23 |
| **DISCUSSION** | | |  |
| Summary of evidence | 24 | Summarize the main findings including the strength of evidence for each main outcome; consider their relevance to key groups (e.g., healthcare providers, users, and policy makers).  The results indicate that a variety of mitigation and control methods for chikungunya have been used worldwide, that a great deal of variability exists in the frequency of use of these mitigation measures, and that there is a lack of consistency among studies when reporting outcomes. | 28, 29 |
| Limitations | 25 | Discuss limitations at study and outcome level (e.g., risk of bias), and at review-level (e.g., incomplete retrieval of identified research, reporting bias).  While there are some limitations to this SR, such as possible language bias due to the exclusion of articles in languages other than English, French, Spanish and Portuguese, or the possibility that research was missed by the scoping review search or not properly classified, every effort was made to minimize potential biases by developing the protocol and all tools used in the scoping review and this SR *a priori* and pre-testing them with the review team. | 31 |
| Conclusions | 26 | Provide a general interpretation of the results in the context of other evidence, and implications for future research.  There was a lack of research that assessed the effectiveness of various mitigation strategies.  With respect to CHIKV research, future studies should focus on consistent reporting of outcomes and the standardisation of how outcomes are measured in order to be able to summarize the effectiveness of individual and complex interventions across studies. | 32 |
| **FUNDING** | | |  |
| Funding | 27 | Describe sources of funding for the systematic review and other support (e.g., supply of data); role of funders for the systematic review.  This research did not receive any specific grant from funding agencies in the public, commercial, or not-for-profit sectors | 32 |

*From:*  Moher D, Liberati A, Tetzlaff J, Altman DG, The PRISMA Group (2009). Preferred Reporting Items for Systematic Reviews and Meta-Analyses: The PRISMA Statement. PLoS Med 6(6): e1000097. doi:10.1371/journal.pmed1000097

For more information, visit: **www.prisma-statement.org**.

Page 2 of 2
